# Multifunctional Fluidic Units for Emergent, Responsive Robotic Behaviors

**DOI:** 10.1002/adma.202510298

**Published:** 2025-11-06

**Authors:** Mostafa Mousa, Alberto Comoretto, Johannes T.B. Overvelde, Antonio E. Forte

**Affiliations:** ^1^ Department of Engineering Science University of Oxford Parks Road Oxford OX1 3PJ UK; ^2^ Department of Engineering Kings College London Strand London WC2R 2LS UK; ^3^ Autonomous Matter Department AMOLF Science Park 104 Amsterdam 1098 XG The Netherlands; ^4^ Institute for Complex Molecular Systems Department of Mechanical Engineering Eindhoven University of Technology PO Box 513 Eindhoven 5600 MB The Netherlands

**Keywords:** implicit synchronisation, pneumatic valves, reconfigurability, self‐oscillating actuators, soft robots

## Abstract

Fluidic circuits have shown significant promise in enabling complex functionality in soft robots with a minimal number of input signals. However, implementing complex behaviors typically involves numerous specialized components, resulting in intricate and nonversatile circuits. To address this challenge, a multifunctional fluidic unit designed to operate flexibly as a valve, sensor, or actuator is introduced. This unit provides an extensive design space that allows precise tuning to achieve the desired functionality. In particular, one configuration integrates all three functions simultaneously, resulting in a self‐sensing oscillating actuator. By assembling multiple units—each customized for specific roles—complex robotic behaviors can be realized. The versatility and effectiveness of this modular approach are demonstrated by creating several robotic systems, including a controlled shaker, a multimodal hopper, and a crawler capable of sensing environmental boundaries. Furthermore, when these units are mechanically coupled via a shared body, it exhibit emergent passive behaviors, such as self‐synchronization—a behavior that is elucidated with a Kuramoto model of networks of oscillators. This study highlights the potential of multifunctionality as a powerful and efficient strategy for realizing embodied intelligence in fluidic robotic systems.

## Graphical abstract

A multifunctional reconfigurable fluidic unit can be used as sensor, valve and actuator is presented. A unique configuration combines the features of the three components as a Responsive self‐oscillating actuator. The remarkable versatility of the fluidic unit is demonstrated by building different robots with the same fluidic units only by varying their arrangement and connections.

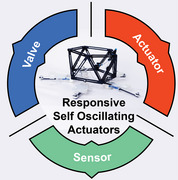

## 1 Introduction

Developments in soft robotics have been influenced by nature and biological systems, providing a new perspective for building today's robots.^[^ ^1^ ^]^ Soft robots benefited from the inherent compliance and adaptability of their structural components, akin to those of their animal counterparts. Such adaptability has proven beneficial for tasks such as navigating irregular terrains^[^ ^2^ ^]^ and versatile manipulation of various objects.^[^ ^3^, ^4^ ^]^ This inspiration and direction toward leveraging the robot body compliance and harnessing the dynamics of its interaction with the environment is known as embodied intelligence.

Recently, fluidic circuits have emerged as powerful tools to enhance physical intelligence in soft robotics.^[^ ^5^, ^6^, ^7^ ^]^ These circuits typically involve interconnected tubing networks,^[^ ^8^, ^9^ ^]^ integrated with mechanical components such as valves,^[^ ^10^, ^11^, ^12^, ^13^, ^14^ ^]^ inflatable actuators,^[^ ^15^, ^16^, ^17^ ^]^ and sensors.^[^ ^18^ ^]^ Strategic combinations of these components enable embedding sophisticated control schemes directly into robotic structures, facilitating complex behaviors like automatic gripping,^[^ ^10^, ^19^ ^]^ actuator sequencing,^[^ ^11^, ^20^ ^]^ and coordinated locomotion,^[^ ^11^, ^21^ ^]^ all without reliance on electronics or software.

However, achieving autonomous environmental interactions typically necessitates numerous specialized components, resulting in scalability challenges. For instance, a locomotor robot capable of autonomously avoiding obstacles typically requires multiple actuators, valves, fluidic sensors, and extensive tubing networks.^[^ ^22^ ^]^

The natural counterparts to soft robots, natural systems, achieve remarkable versatility by assigning multiple functionalities to the same body part.^[^ ^23^ ^]^ For example, plant roots simultaneously provide anchorage, nutrient uptake, and environmental sensing.^[^ ^24^, ^25^ ^]^ Similarly, elephant trunks facilitate feeding and breathing, while also enabling manipulation,^[^ ^26^ ^]^ sensing,^[^ ^27^ ^]^ and olfaction.^[^ ^28^ ^]^ Animals often employ their tongues not only for feeding and communication,^[^ ^29^ ^]^ but also for sensing^[^ ^30^ ^]^ and grooming.^[^ ^31^ ^]^ Following a similar strategy to embody autonomy, there is ample room for investigation of multifunctional, versatile fluidic devices for a wider range of responsive behaviors.

To address this challenge, researchers have developed multifunctional fluidic units, most of which are variants of valves. Some valve designs have been augmented with self‐sensing features, proving useful in safety‐related applications^[^ ^32^ ^]^ and wearables.^[^ ^33^ ^]^ Other approaches have integrated switching mechanisms into soft actuators, thereby enabling multimodal locomotion.^[^ ^34^ ^]^ This development builds on the concept of embedded control in soft robots, where valves are incorporated into the robot body and configured into oscillator circuits that drive actuation for locomotion.^[^ ^19^, ^21^ ^]^ Along these lines, we previously introduced a fluidic oscillator that simultaneously serves as a robotic limb.^[^ ^35^ ^]^ Despite such advances, the expanding library of fluidic components still lacks a truly multifunctional unit that can operate as a valve, sensor, and actuator. The availability of such a unit not only broadens the design space of soft robots beyond task‐specific components, but also provides direct feedback from the environment, enabling its use as a platform for decentralized physical control in fluidic robots.

Inspired by nature's approach to multifunctional body parts, we propose a modular fluidic unit capable of reconfigurable, multifunctional operation, allowing diverse soft‐actuated robots to be constructed with minimal hardware changes. By selectively activating or deactivating ports, a single fluidic unit can flexibly function as a sensor, an actuator, and a valve, or even as self‐sensing oscillating robotic limbs. These limbs, besides combining the features of the three configurations (sensing, actuation, and oscillatory behavior), implicitly synchronize^[^ ^35^ ^]^ their oscillations in specific patterns when tessellated in different geometries. We elucidate these patterns of oscillations using a model based on Kuramoto,^[^ ^36^, ^37^ ^]^ which is widely used for describing synchronization in networks of oscillators.^[^ ^38^ ^]^

Exploiting the versatility of our units, we demonstrate various oscillating circuits, including relaxation and ring oscillators, and illustrate their seamless integration with units performing sensing and flow regulation. Ultimately, mounting and reconfiguring these multifunctional units on a robotic skeleton enables the realization of distinct robotic systems—a shaker, a hopper, and a boundary‐sensing crawler—each showcasing emergent behaviors arising from different configurations of the same foundational unit.

## 2 Results

### 2.1 Fluidic Unit Operation

**Figure 1:**
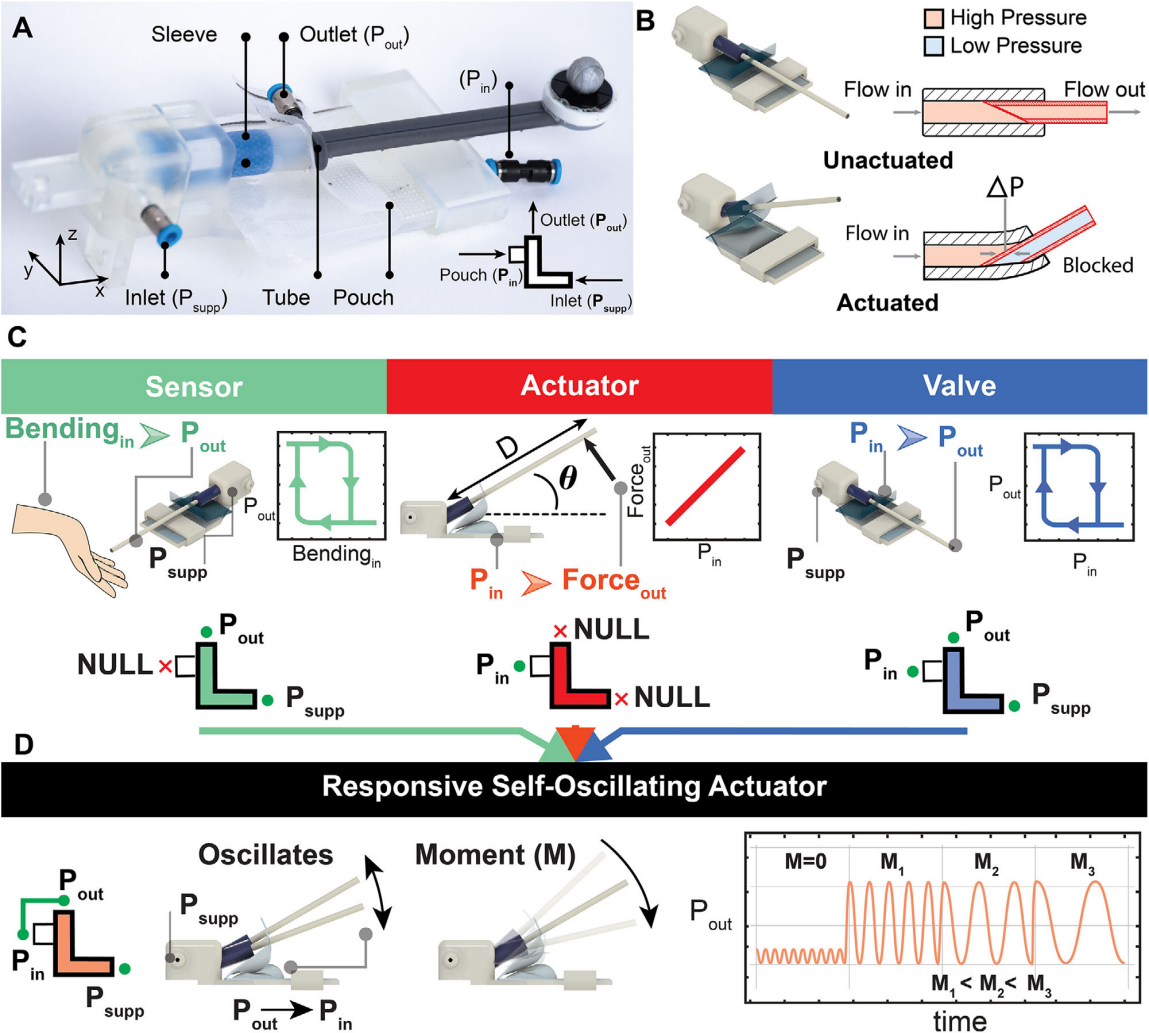
Fluidic Unit Operation. A) The fluidic unit and its main components. B) Rendered images of the unit in its unactuated and actuated states, with an illustration of the sleeve cross‐section showing flow pressure in both cases. C). The unit is configured as a sensor, actuator, or valve, with corresponding input/output connections. The middle panel shows the bending angle (*θ*) and the moment components, where *M* = *F × D*. D). The responsive self‐oscillating actuator symbol and connection. Once pressurized, it oscillates at high frequency with small amplitudes; when an external moment is applied, the oscillations increase in amplitude and decrease in frequency.

The fluidic unit (**Figure** **1**A) expands upon a soft‐sleeve valve that we initially developed for frequency control in fluidic oscillators.^[^ ^14^ ^]^ The primary components of this unit, depicted in Figure 1A, include a valve body, a silicone sleeve, a 3D‐printed tube, and a pneumatic actuator (pouch). Depending on the intended application, the inlet, outlet, and pouch input of the unit may be utilized or left disconnected.

In this design, a tube nested inside a compliant silicone sleeve is bent until its tip presses against the sleeve's inner wall, blocking the passing airflow inside the tube itself (Figure 1B; Movie S1, Supporting Information). Additional details on the operation, fabrication, and materials of the unit are provided in the Experimental Section. The unit is highly versatile: the nature and arrangement of its components allow it to be reconfigured into distinct fluidic devices (Figure 1C).

First, the unit can be configured as a mechanical sensor that triggers or blocks a fluidic signal when mechanically actuated.^[^ ^22^ ^]^ This configuration is analogous to insect antennae, which are densely packed with mechanosensory structures.^[^ ^39^ ^]^ To function as a sensor, the unit is connected to airflow through its inlet and outlet ports; when the tube is pushed upward and the sleeve bends, the airflow is blocked.

Second, the unit can be configured as an actuator. Unlike the sensor setup, this configuration disregards the inlet and outlet ports and relies solely on the pouch. Inflating the pouch generates a moment that rotates the tube‐sleeve assembly around the unit's rigid enclosure. This moment can then be harnessed to lift a weight or even enable locomotion, as demonstrated in later sections of the manuscript.

Third, complementing the sensor and actuator configurations, the unit can operate as a pneumatic valve. When its inlet and outlet are connected to airflow, pressurizing the pouch bends the sleeve, thereby blocking the flow. As the pouch pressure decreases, the sleeve's restoring moment returns the unit to its idle position, reopening the passage. Experimental characterization of the valve switching (Figure S1, Supporting Information) reveals strongly hysteretic behavior. Several factors contribute to this hysteresis, including the nonlinearity of the sleeve material and the intrinsic hysteresis of the pouch, but we believe the dominant source is the switching mechanism itself, namely, sleeve bending and inner‐tube rotation. Once the tube rotates to block the signal, a pressure difference (∆*P*) develops between the inner and outer sides of the tube (Figure 1B). This Δ*P* stabilizes the blocked state even after pouch pressure falls below the switching‐on threshold, resulting in switching off at a lower pressure.

Last, we explore a unique configuration where the fluidic unit exhibits the features of sensing, actuation, and switching combined (Figure 1D). By connecting the unit's outlet to the pouch, it exhibits an oscillatory behavior where it periodically switches from open to closed states. These oscillations are magnified in magnitude once the tube is opposed by another force, in other words, when opposed by a counter moment. Interestingly, the oscillation frequency scales with the external moment as well. This configuration will be characterized in detail and used for functional applications in the following section.

A key advantage of the unit's architecture is its large, tunable design space: by varying the geometry or material of the components, one can fine‐tune its blocking threshold and actuation behavior. We systematically explore three parameters—sleeve material, tube cut profile, and pouch geometry (Figure S1A, Supporting Information)—and quantify their influence on the unit operation in its three configurations; sensor, actuator, and valve in the supplementary materials (Figure S1B, Supporting Information). Although our parametric study reveals a broad design space, to maintain experimental consistency and clearly demonstrate the fluidic unit's capability to realize multiple robotic configurations with minimal hardware variation, we fix the design parameters for all subsequent experiments. The selected configuration includes a DragonSkin 30 silicone sleeve, an angled flat‐cut outlet tube, and a two‐segment polyethylene (PE) pouch actuator (Figure S1B, Supporting Information).

### 2.2 Responsive Self‐Oscillating Actuator

Fluidic oscillators offer an ideal strategy for enabling electronic‐free soft‐actuated robots to achieve autonomous behavior. We explore the unit's capability to construct two types of oscillatory circuits: relaxation oscillators and ring oscillators (Figures S2 and S3, Supporting Information). We also investigate how these circuits behave when connected to sensor and actuator units. We demonstrate this integration by building a controlled shaker that can dispense beads in desired containers based on user input (Figure S4 and Movie S3, Supporting Information).

**Figure 2:**
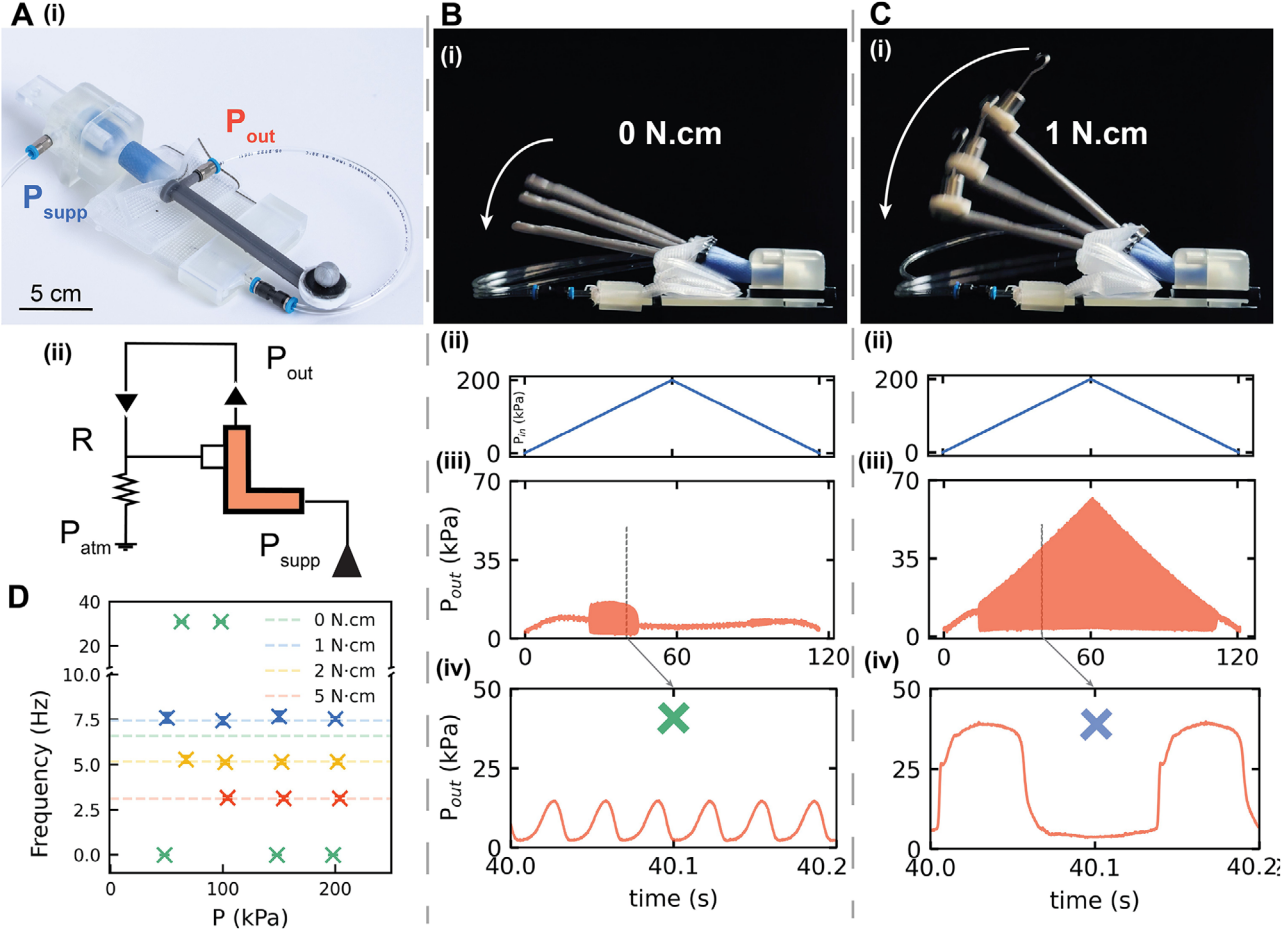
Fluidic Unit as Self‐oscillating Actuator. A) A photo of the fluidic unit in a relaxation oscillator circuit, with its outlet connected to its inlet (A‐i), and a schematic illustrating the direction of flow and leakage to the atmosphere (A‐ii). B) A photo of the fluidic unit showing the position of its tube during operation as a relaxation oscillator, before any countermoment is applied (B‐i). The unit is tested by sweeping the input pressure from 0 to 200 kPa (B‐ii). In this configuration, the unit exhibits oscillatory behavior over a limited pressure range (B‐iii), with a notably high oscillation frequency (B‐iv). C) When a small countermoment is applied, the stroke length of the tube increases (C‐i). Under the same pressure sweep (C‐ii), the unit now exhibits consistent oscillations across the full pressure range (C‐iii). These oscillations maintain a constant frequency, while their amplitude increases proportionally with input pressure. However, this consistency comes at the cost of a reduced oscillation frequency compared to the case without a countermoment (C‐iv). D) There is an inverse relationship between the applied countermoment and the oscillation frequency: the higher the countermoment, the lower the resulting frequency. The mean of five experiments is shown as a dashed line, while the standard deviation is represented by error bars.

In this section, we analyze the unit's oscillatory behavior and demonstrate how a single configuration can yield fast, reliable, and sensitive self‐oscillating actuators. The design is based on a relaxation oscillator circuit: the fluidic unit's inlet is connected to a constant pressure supply, the outlet serves as the valve output, and the pouch acts as the valve input. To complete the circuit, the outlet is connected both back to the pouch and to ground via an embedded leak to atmospheric pressure (Figure 2A (i,ii)).

The valve's oscillations arise from alternating charging and discharging phases.^[^ ^14^ ^]^ First, during charging, airflow travels from the inlet to the outlet, pressurizing the pouch until a critical threshold is reached; at that moment, the valve switches from open to closed, and the pressure waveform rises from its minimum to its maximum. Next, in the discharging phase, inlet flow ceases and the pouch releases its air volume to the atmosphere via the pneumatic leak. Because of the valve's hysteresis and the fluidic resistance, the pouch does not immediately repressurize; instead, the outlet pressure falls back to a lower threshold, at which point the valve reopens, a new cycle begins, and then it keeps oscillating (Figure 2B (i)).

The behavior of this circuit is highly sensitive to both input pressure and fluidic resistance. When sweeping the supply pressure (Figure 2B (ii)), oscillations occur only within a limited range (≈75–140 kPa) (Figure 2B (iii)). Despite the fact that these oscillations have a very high frequency (31 Hz), they are not evident at higher pressures, and their amplitude is not proportional to the pressure input (Figure 2B (iv)). Although higher pressures can induce oscillation with flow regulators (i.e., when one embeds the unit into a circuit), we sought a purely mechanical solution to preserve minimal hardware.

The two phases of oscillation are governed by competing moments: pouch inflation generates a moment about the unit hinge that closes the valve, while the sleeve's elasticity produces a counter‐moment that resets the pouch and reopens the flow path. However, at high pressures, the discharge flow can re‐pressurize the pouch before it fully resets, causing it to stall in an intermediate state. Therefore, an additional force is needed to increase the sleeve's restoring moment. In order to demonstrate this, we add a counterweight at the tube tip (Figure 2C (i)).

Consequently, when sweeping the pressure input, the unit maintains fast, consistent oscillations, with a stable frequency even as pressure rises (Figure 2C (ii,iii)). The pressure sweep now demonstrates reliable functionality up to 200 kPa, and the oscillatory behavior persists at input values as high as 400 kPa. This comes at the cost of reducing the output frequency compared to the previous case, where no counter moment was applied (Figure 2C (iv); Movie S4, Supporting Information). Increasing the counterweight shifts the value of the input pressure at which the oscillations start, and decreases the oscillation frequency further (Figure 2D, average and standard deviation), which stays constant for any value of *P_in_*. This is expected as in this configuration, the pouch requires higher pressure to reach the bending moment required to block the flow.

The results presented here illustrate the multifunctional capability of the fluidic unit. Initially, the fluidic unit is configured as a valve within a relaxation oscillator circuit. This configuration yields oscillations that are responsive to external stimuli, specifically the applied counterweight or counter moment. Additionally, when subjected to an external force, the displacement generated at the tip of the tube increases significantly (as demonstrated in Figure 2B (i),C (i)), highlighting its potential use as an actuator, as further detailed in subsequent sections.

### 2.3 Soft Hoppers that Synchronize Their Limbs

Robotic hoppers are a challenging type of locomotor to build using soft actuators. Hoppers require rapidly developing force upon ground contact, which must be applied periodically to sustain continuous hopping. However, soft inflatable actuators typically suffer from slow response times.^[^ ^40^ ^]^ In previous work,^[^ ^14^ ^]^ we developed a soft hopper driven by a relaxation oscillator. However, that design was fixed to a seesaw mechanism and required a counterweight to synchronize the hopper's time of flight with the oscillator frequency.

Here, we utilize our responsive self‐oscillating actuators as robotic limbs for a soft hopper. As seen in the previous section, these actuators are self‐sensing units, making them well‐suited for the hopper's need to actuate upon ground contact. They generate sufficient force to lift the hopper off the ground, and because they are configured in an oscillatory circuit, they continue providing a cyclic output as long as we provide constant pressure as input. These characteristics make them ideal candidates for use as hopping limbs.

**Figure 3:**
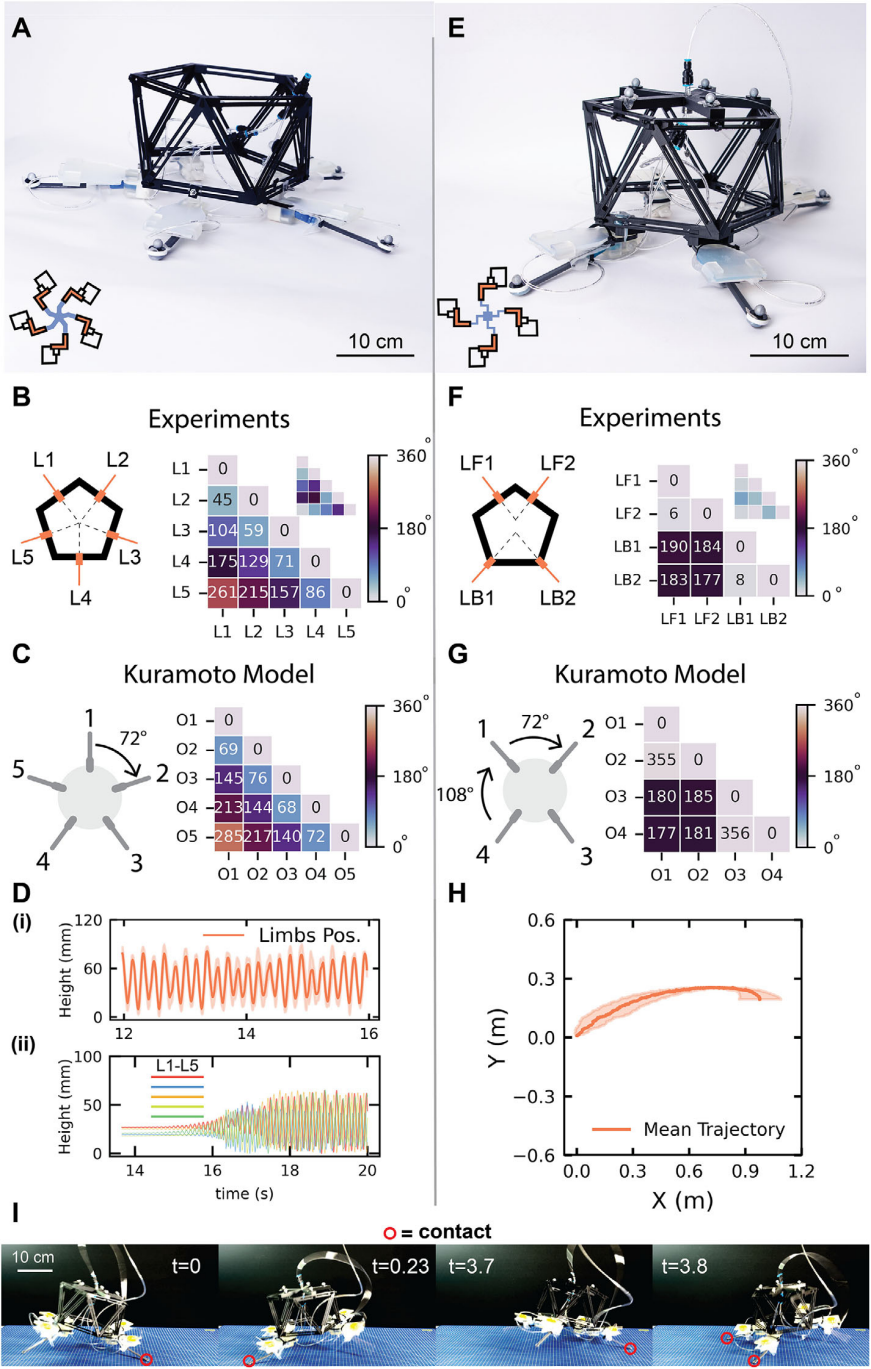
Soft Actuated Hopper. A) A photograph of the five‐limb hopper and a schematic of the hopper's pneumatic circuit (bottom left). B) The spatial distribution of limbs on the icosahedron‐shaped body, and a heatmap showing the phase differences in degrees between each limb and all others, where 180° is anti‐phase, and 0–360° is in‐phase. In the top‐right corner, an inset shows the heatmap of the limbs’ phase difference when operating off the ground. C) Illustration of the arrangement of the units as defined in the Kuramoto model, and a heatmap showing the predictions of the phase differences matching the experimental results. D‐(i)) The mean height reached by each limb over a four‐second time window, and D‐(ii)) the evolution in time of the limbs’ height from the start of the experiment, showing the emergence of the hopping behavior at the critical pressure. E) A photograph of the four‐limb hopper and a schematic of the corresponding fluidic circuit (bottom left). F) The distribution of limbs on the icosahedron body, and a heatmap illustrating the passive synchronization between the front and back limbs. The inset in the top right corner shows a low phase shift between the limbs before touching the ground. G) Illustration of the orientation of the units as defined in the Kuramoto model, and a heatmap showing the predictions of the phase differences matching the experimental results. H) The mean trajectory followed by the forward‐locomoting robot. I) Snapshots of the four‐limb hopper in E at different time stamps, displaying the galloping gait of the robot.

We employ the same hollow icosahedron structure used in the shaker setup (Figure S8, Supporting Information), along with the same fluidic units (**Figure** **3**A). Five fluidic units are arranged in a pentagonal configuration at the base, forming the hopper's five limbs. This symmetric configuration ensures that each resultant force from the interaction with the ground passes through the center of the robot. The limbs are flipped to face downward, and all are powered by a single constant pressure source (610 kPa) (Figure 3B).

Upon pressurization, the hopper initially exhibits a transient period of random limb actuation. Eventually, a coordinated behavior emerges, and the robot begins to hop continuously as the limbs actuate alternatively in succession. Phase difference heatmaps confirm this coordination: adjacent limbs (e.g., L1 & L2, L3 & L4) exhibit low phase differences, while limbs on opposite sides (e.g., L1 & L3, or L4) show higher phase differences (Figure 3B). In particular, the phase differences show that the limbs activate in succession. For instance, focusing on limb L1 in Figure 3B, we see that limb L2 will activate with a phase of *≈*45°, then L3 with *≈*104°, then L4 with *≈*175°, and lastly L5 with *≈*261°. This behavior only emerges when the limbs are coupled with the ground, as demonstrated via the insets in Figure 3B, where the robot is operating off the ground.

### 2.4 A Kuramoto Model of Implicit Coupling

Following the experimental observation of such temporal patterns, we aim to gain a deeper understanding of this synchronization phenomenon. Synchronization of oscillators is widely observed in nature,^[^ ^41^ ^]^ from swarms of fireflies that together synchronize their firing to attract females for mating,^[^ ^42^ ^]^ to the pacemaker cells of a heart to produce patterned beats.^[^ ^43^ ^]^ The Kuramoto model^[^ ^36^, ^37^ ^]^ is widely used to model these coordinated patterns in different domains. Here, we develop a basic model that builds upon Kuramoto to investigate the robot's hopping pattern. With the model, we aim to test the hypothesis that synchronization is a consequence of the implicit coupling between the units, facilitated by the common robot's body and by the shared substrate, analogous to our previous experimental work on physical synchronization.^[^ ^35^ ^]^ Moreover, we aim to capture how different arrangements of the units on the robot's body affect the various distinct synchronization patterns that emerge.

We report the extensive model derivation in the supplementary materials (Figures S10 and S11, Supporting Information). In summary, we build on the standard Kuramoto model, for which the phase of each oscillator *i* coupled to another oscillator *j* follows:^[^ ^36^, ^37^ ^]^

(1) $$\frac{d\theta_{i}}{dt}=w_{i}+\sum_{j=1}^{N}K_{ij}\sin\left(\theta_{j}-\theta_{i}\right)$$

where *ω_i_* indicates the natural frequency of oscillator *i*, *θ_i_* and *θ_j_* the phases of the two oscillators, and *K_ij_* the coupling constant between the two oscillators.

Building on this model, we make a single, key assumption: the relative orientation between two units on the robot's body plan determines the sign and magnitude of the coupling constant between that pair of oscillators (Figure S10, Supporting Information). This is a consequence of the implicit nature of the coupling, that arises from the shared substrate and the common robot's body. We assume that the oscillators are positively coupled when aligned in parallel (as they promote each other by lifting the shared body), or negatively coupled if opposite to each other (as lifting the body inhibits the other oscillator) (Figure S10, Supporting Information). This assumption leads to a relatively simple remapping of the relative orientation between each pair of units *β_ij_* and their corresponding coupling constant *K_ij_*:

(2) $$K_{\mathrm{ij}}=\cos\;\beta_{ij}$$

This assumption alone allows us to recapitulate the phase shift between units observed experimentally (compare Figure 3B with Figure 3C), given an arbitrary number of units and orientations (Figure S11, Supporting Information).

### 2.5 Tuning Synchronization for Forward Locomotion

Although the limbs on the robot achieve an average vertical displacement of up to 70 mm (Figure 3D (i,ii)) with *≈*6 hops each second, this specific configuration does not lead to forward locomotion, since the limbs are arranged uniformly and symmetry is not broken. Hence, to achieve forward locomotion for functional robotic applications, we intentionally break the symmetry of the limb layout to introduce a directional force bias (Figure 3E). In this new design, the hopper has two back limbs (LB1 & LB2) and two front limbs (LF1 & LF2). The back limbs are positioned at the base vertices, while the front limbs are placed opposite them but shifted inward by 10 mm to create the necessary asymmetry (Figure 3F).

This new configuration results in a distinct behavior. After a transient period, the limbs self‐organize such that i) the back limbs actuate in‐phase with each other, and likewise for the front limbs, and ii) the two pairs actuate in anti‐phase with each other. This coordinated pattern is clearly visible in the phase difference heatmap (Figure 3F). Excitingly, our Kuramoto model predicts that such a minor change in the arrangement of the limbs on the robot's body leads to this largely different synchronization pattern (Figure 3G). Moreover, the model predicts that, theoretically, other arrangements could lead to similar patterns: for instance, two triplets can oscillate anti‐phase with each other, while the oscillators within each triplet are in‐phase (Figure S11, Supporting Information).

As a result of the asymmetrical design, the hopper begins to move forward—despite minor directional deviations due to assembly imperfections (Figure 3H,I). This version of the hopper locomotes at a speed of *≈* 0.3 BL.s*^−^* ^1^ (Movie S5, Supporting Information).

### 2.6 Crawler Sensitive to End of Terrain

Embedding fluidically driven robots with decision‐making capabilities typically requires the integration of multiple components. A hysteretic element is needed to convert a single input into alternating outputs that drive the robot's actuators. An element sensitive to external stimuli is required to input information into the system. Finally, a control element is needed to switch the robot from one state to another. Fortunately, all these functionalities can be realized using our fluidic unit.

**Figure 4:**
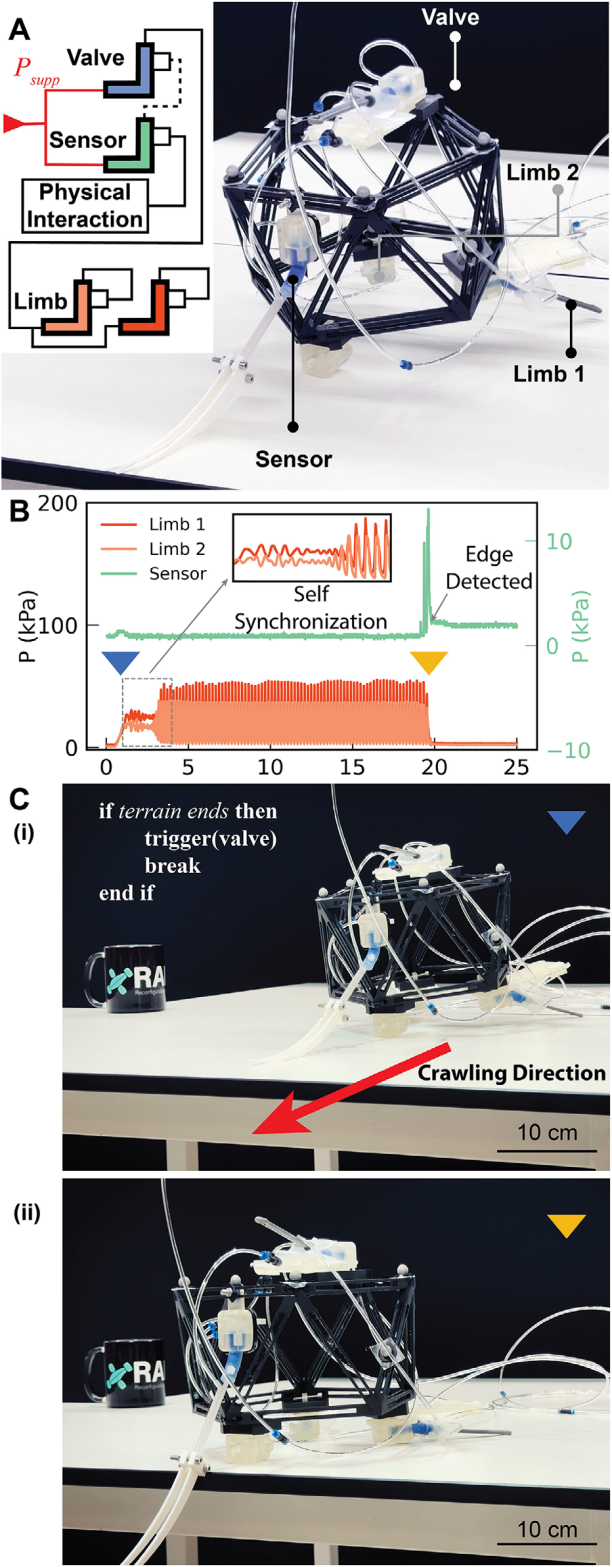
Crawler Operation. A) A photo of the robot with its units labeled and the circuit schematic in the top left corner. B) A plot showing the different phases of the crawler operation; initial passive synchronization magnified in the inset, oscillation of the limbs, and finally the sensor detecting the edge of the table and stopping the limbs from further locomotion. C) Snapshots of robot locomotion at the start (i) and end (ii), with the programmed behavior specified in IF–THEN format in the top left corner.

Here, we build a crawler that can detect the end of the terrain it is traversing. To achieve this, we use four fluidic units configured for different roles. The first acts as a sensor, the second as a valve, and the remaining two as self‐oscillating actuators. The sensor unit is pre‐actuated, placed so that its sleeve is bent under the robot's weight against the table. The valve functions as a safety interlock; when triggered by the sensor's output, it halts the pressure to the rest of the circuit. The two self‐oscillating actuators serve as the robot's limbs, autonomously pushing the robot forward (**Figure** **4**A). In line with the pseudocode representation introduced in,^[^ ^44^ ^]^ this behavior can be formulated as an IF–THEN statement: IF the terrain is absent, THEN the valve cuts off the flow to the actuators. To demonstrate the versatility of our approach, the robot body and fluidic units are the exact same ones used in the previous sections, where two units that were configured as self‐oscillating actuators are now reconfigured to be a valve and a sensor.

When pressure is provided as input to the robot, air flows through the safety valve to the limbs. Initially, the limbs oscillate out of phase, but they gradually synchronize, producing coordinated movements that propel the robot forward. The robot continues this forward motion until it reaches the edge of the table. At that point, the sensor—which was previously held closed by the table's surface—opens, allowing air to enter the safety valve pouch. The valve then actuates, blocking airflow to the limbs and stopping the robot, preventing it from falling (Figure 4B,C; Movie S6, Supporting Information).

The robot underwent testing both while in contact with the ground and while suspended. Before contacting the ground, the limbs oscillated at high frequency, with the tube tip exhibiting only a small stroke. Upon ground contact, the limb motions were initially uncoordinated and actuated in a seemingly random manner. Over time, however, they passively synchronized, ultimately resulting in coherent forward motion (Figure S9, Supporting Information).

## 3 Conclusion

Fluidic control holds great potential for creating more adaptive and responsive systems, with minimal inputs. Here, we introduce a fundamental pneumatic fluidic unit that can be configured in different ways to function as a valve, sensor, or actuator. By arranging and connecting multiple identical units in a specific manner, we can construct a responsive, pneumatically actuated soft robot. This approach significantly reduces the complexity of control circuit design. Instead of relying on multiple distinct components to build a control circuit for a specific actuator, a single fluidic unit is sufficient to serve as a switching element, oscillator, environmental interface, and actuator.

The unit can also combine all the features of control, sensing, and actuating at once. When configured as a self‐oscillating actuator, it exhibits distinct oscillation patterns compared to standard actuation modes, with its frequency determined by the applied load. By incorporating multiple self‐oscillating actuators as robotic limbs, an emergent collective behavior passively arises due to the coupling of these units through the robot's body structure. Owing to their ability to sense and respond to opposing bending moments, the units can communicate implicitly through mechanical interaction. This implicit communication facilitates their synchronization—a behavior that would likely not emerge in traditional paradigms, where units are task‐specific and coupled only through fluidic, not physical, connections.

However, these advantages come with certain challenges. While the fluidic unit efficiently performs its intended functions, its fabrication process is time‐consuming and involves multiple components. One key area for improvement is investigating methods to manufacture the cell in a monolithic form.^[^ ^19^ ^]^ Additionally, a software framework is needed to organize and integrate these cells effectively. Transforming the fluidic units into a voxel‐like format—allowing them to interconnect while maintaining their functionality—would enhance the practicality and ease of use of this design.

One of the motivations for embodying responsive behaviors through versatile units is the potential to physically scale down robots to the microscale without sacrificing functionality. The mechanics of our devices are, in principle, scale‐independent, as they are governed primarily by geometry and elasticity. Fluidics, however, are inherently scale‐dependent. At the macroscale (as in this work), the flow is turbulent with a high Reynolds number (≈7000), both within the fluidic circuits and in the surrounding air. As these circuits are scaled down, the Reynolds number decreases, and viscosity can be dominant over inertia, both internally and in external interactions. Importantly, previous demonstrations of microvalves in microfluidics suggest that control and gating remain feasible at this scale.^[^ ^45^, ^46^ ^]^ Nevertheless, this raises an open question for future work: how can implicit coupling be achieved in a surrounding medium at low Reynolds numbers?

Physical and mechanical intelligence approaches facilitate the development of adaptive, responsive, and environmentally aware robots. By encoding control strategies directly into the robotic body, these systems can offload significant computational demands from high‐level controllers. This approach not only enables the development of intelligent, electronics‐free robots, but also enhances the efficiency of electrically driven robots by shifting many functional responsibilities to the mechanical structure.

## 4 Experimental Section

### Fluidic Unit Fabrication

The fluidic unit consisted of four main components: the unit body, the sleeve, the tube, and the pouch (Figure S5, Supporting Information). Both the valve body and the tube were 3D‐printed using the Formlabs Form 3B+ SLA printer. The body was printed using Formlabs Clear Resin, while the tube was fabricated with Formlabs Tough 2000 Resin for enhanced durability. Internal air channels were incorporated into both parts; to prevent blockage during post‐curing, the channels were thoroughly flushed with isopropyl alcohol using a syringe and then dried with compressed air. Post‐curing was performed according to Formlabs’ standard protocols to ensure optimal material properties.

Throughout the experiments described in this manuscript, Festo 3 mm PE flexible tubing was used for all pneumatic connections. These tubes were press‐fitted into the inlet and outlet ports of the valve body and the tube, respectively. Similarly, the flexible sleeve was press‐fitted onto the 3D‐printed tube, which featured an internal channel extending from the inlet port.

The plastic pouches were fabricated from commercially available PE Latern vacuum sealing bags on Amazon. Each pouch was divided into multiple segments, each measuring 70 × 50 mm. To allow controlled leakage to the atmosphere, each segment included six holes, 1.3 mm in diameter, serving as resistive leak points. These holes were arranged in two horizontal rows—three on each side of the segment—spaced 10 mm apart. They were punch‐pressed using a Metcal straight dispensing tip. The pouch was initially sealed on three sides to form a rectangular enclosure, leaving one short side open for tube insertion. The total sealed length equaled 70 mm multiplied by the number of segments, with a fixed width of 50 mm (Figure S6, Supporting Information).

The Festo 3 mm tube was inserted ≈20 mm into the open side of the pouch, and glue was applied to a depth of about 10 mm to ensure an airtight seal. An additional 10 mm section was then sealed at the top to create a “bleed” area, which reduced the internal volume of the pouch. This bleed contained a small hole through which the outlet tube was inserted, securing the pouch's position between the tube and the sleeve. Finally, the pouch was folded into a zigzag pattern so that the segments stacked vertically, and the folded structure was inserted into a dedicated holder within the valve body.

### Fluidic Unit Characterization Experiments

The effects of material choice and geometric parameters on the fluidic unit's operation were investigated through a series of characterization experiments. These tests examined the threshold bending moment or angle at which airflow was obstructed, depending on the unit configuration.

The fluidic unit was mounted in an Instron 68TM‐50 tensile testing machine equipped with a 500 N load cell (Figure S7A, Supporting Information). A custom 3D‐printed base was fitted inside the machine to position the unit so that the outlet tube aligned vertically with the load cell. A fork‐shaped 3D‐printed attachment was mounted on the load cell, holding a V‐groove SKF bearing between its prongs. This fork‐bearing assembly allowed the tube to slide smoothly with minimal friction while being pushed upward during testing (Figure S7B, Supporting Information).

Test control and data acquisition were carried out using Bluehill Universal software. A standard tensile test was performed at a displacement rate of 0.1 mm s^−1^. The zero position was defined as the point where the tube just contacted the bearing. Data were collected at 100 Hz. The data were then exported in CSV format for further processing.

Airflow was supplied to the fluidic unit, and pressure changes were recorded using a Panasonic ADP5150 analog pressure sensor. The sensor was connected to a National Instruments (NI) USB‐DAQ 6001, and the data were logged using NI DAQExpress software, also at a 100 Hz sampling rate (Figure S7C, Supporting Information).

For the pouch force characterization experiment, the fork attachment was replaced with a straight connector. The machine head constrained the tube from moving upward, and the pouch was then pressurized. The maximum force at zero displacement was recorded. Next, the Instron head moved upward, allowing the tube to rotate around a hinge. This setup enabled measurement of the force decay as a function of the unit's bending angle.

Finally, a separate valve characterization experiment was conducted using only a pressure controller. The ElveFlow OB1 pressure controller was used to sweep the pressure inside the pouch, while a constant pressure supply was applied at the valve inlet. The outlet was connected to a Festo throttle valve, serving as a fluidic resistor vented to the atmosphere and connected to the analog pressure sensor. All these tests were carried out five times to ensure consistency and to calculate the standard deviation.

### Robot Locomotion Tracking

The movements of the robots and their self‐oscillating limbs were recorded using a Vicon motion tracking system. This system consisted of 10 Vero cameras arranged around the robot's operating area on a supporting frame. The cameras were connected to a central hub that streamed real‐time data to a dedicated computer, where the position data was logged using Tracker software.

To capture the overall position of the robot as well as specific components such as the self‐oscillating limbs, passive infrared markers were attached to various points on the robot's body at non‐symmetrical, random locations and to each limb tip. Avoiding symmetrical marker placement was important to prevent the software from misidentifying the robot's orientation. The software allowed the whole robot to be tracked as one object by linking the markers, and it also made it possible to track each marker individually. This allowed the tracking of both robots’ overall movement and the motion of each limb.

## Conflict of Interest

The authors declare no conflict of interest.

## Supporting information



Supporting Information

Supplemental Movie 1

Supplemental Movie 2

Supplemental Movie 3

Supplemental Movie 4

Supplemental Movie 5

Supplemental Movie 6

## Data Availability

The data that support the findings of this study are available from the corresponding author upon reasonable request.
