# Supplementary material for: Multifunctional Fluidic Units for Emergent, Responsive Robotic Behaviors
Source: Adv Mater. 2025 Nov 6;38(16):e10298. doi: 10.1002/adma.202510298 (PMC12994330; doi:10.1002/adma.202510298)
Supplement: Supplementary file 1 — Supporting Information [file ADMA-38-e10298-s006.docx]

# Supplementary materials

Controlled Relaxation Oscillator Controlled Ring Oscillator Controlled Soft Actuated Shaker

Model of the Emergent Synchronization from Implicit Coupling of Units Movies Captions S1 to S6

Figures S1 to S11 Movies S1 to S6

## Fluidic Unit Design Space

In this section we assess the effect of changing different material and geometrical parameters on the fluidic unit operation. The rich design space of the fluidic unit enables designers to tune the fluidic unit for specific applications. We mainly dis- cuss the possibilities of changing the silicone sleeve material, the tube cut profile, and the pouch design (Fig. S1A).

The soft silicone sleeve must be sufficiently flexible to bend without ballooning under internal pressure. To meet these criteria, we embed an inextensible fabric core within commercially available silicone rubber. As the sleeve functions as a hinge within the valve body, its shore hardness directly governs hinge stiffness, with increased hardness corresponding to increased stiffness. To systematically investigate this relationship, we test three silicone rubbers of different shore hardness levels (10A, 20A, and 30A). In this experiment, the unit operates in the sensor mode: a constant pressure (*P_supp_*) is sup- plied to the inlet, outlet pressure (*P_out_*) is measured, and the pouch input (*P_in_*) remains disconnected. The tube is incre- mentally bent using a tensile testing machine, with resistance force measurements taken throughout. Experimental results confirm a clear relationship between increasing hinge stiffness and higher bending moments and angles required to inter- rupt airflow (Fig. S1B (i)).

Flow blockage occurs when the outlet tube’s end presses against the silicone sleeve. Previous study indicate that the tube’s cut angle significantly influences the required bending moment to achieve blockage (14). To further characterize this ef- fect, we fabricate two distinct outlet tubes via 3D printing: one with a flat-cut profile and another with a curved-cut pro- file (Fig. S1A). Experiments comparing these profiles show that the curved-cut tube consistently requires higher bending moments and angles to achieve flow blockage compared to the flat-cut tube. Furthermore, while the bending threshold for the curved-cut profile remains stable across different flow pressures, the flat-cut tube exhibits a proportional increase in the required bending moment and angle with higher flow pressures (Fig. S1B (ii)).

The force necessary to bend the outlet tube is provided by a sealed polyethylene pouch, pre-folded in a zigzag pattern. Upon inflation, the pouch unfolds, exerting a force that pushes the tube into the silicone sleeve. We assess pouch perfor- mance by comparing two folding configurations: a two-segment and a three-segment pouch. We start by characterizing the unit’s performance as an actuator by connecting only the pouch input (*P_in_*). At three different constant inflation pressures, we measure the bending force required as a function of bending angle (applied under displacement-controlled tensile test- ing; Experimental Section). The three-segment pouch—with its larger internal surface area—generates greater force at the same inflation pressures when compared to the two-segments pouch (Fig. S1B (iii)).

Next, we assess the valve configuration by applying a constant supply pressure (*P_supp_*) to the inlet and varying the pouch pressure (*P_in_*) to determine the threshold at which airflow to the outlet ceases. Consistent with previous findings, the three- segment pouch requires lower internal pressures to block airflow, thus lowering the overall operational thresholds of the valve (Fig. S1B (iv)).

## Controlled Relaxation Oscillator

Oscillator circuits are popular to equip robots with autonomy. As they produce repetitive, periodic signals that can be used to drive the robot actuators. Relaxation Oscillators are one simple type of oscillators where one inverter (valve) is used to generate oscillations. In this circuit, the valve, outlet is connected to its input (pouch) and to a leak to the atmo- sphere. In this section we explain this circuit operation, and how it changes when coupled with other fluidic unit in sensor configuration.

The oscillatory behavior exhibited by the valve can be understood by examining its charging and discharging phases. In the charging phase, airflow passes from the valve inlet to its outlet, pressurizing the valve’s actuator (pouch) until a critical pressure threshold is reached. At this point, the valve switches from its open to closed state. This switching event marks the end of the charging phase, during which the pressure waveform rises from its minimum to maximum value.

During the discharging phase, the flow that initially pressurized the pouch and triggered switching is no longer present. The pressurized pouch can now release the entrapped air volume to the atmosphere without being replenished by flow from the inlet. This reset is only possible due to the hysteretic nature of the valve and the presence of pneumatic resistance;

otherwise, the pouch would be instantly re-pressurized, stabilizing at an intermediate state. In this phase, the pressure at the outlet drops back to a critical threshold. Once this minimum threshold is reached, the valve switches back to its open state, completing one full oscillation cycle and initiating the next.

As mentioned, we pair the relaxation oscillator circuit with an additional fluidic unit configured as a sensor (Fig. S2A). In this configuration, the fluidic unit’s inlet and outlet act as the sensor’s two ports, allowing it to be integrated into a cir- cuit in series, similar to a resistor in an electrical circuit. The pouch is left unconnected and unused in this setup, enabling physical interactions with the tube to induce rotation around the hinge, thereby altering airflow between the inlet and out- let.

When the bending angle of the sensor cell changes, both the frequency and amplitude of the output pressure vary (Fig. S2B). This relationship remains proportional as long as flow continues, until a threshold is reached where the sensor fully blocks airflow in the circuit. The change in frequency is most noticeable within a narrow bending angle range (28–32°) (Fig. S2E), aligning with the fluidic unit characterization discussed earlier.

## Controlled Ring Oscillator

We continue our investigation of pairing oscillator circuits with a fluidic unit in a sensor configuration. In this section, we focus on the ring oscillator circuit. A ring oscillator is a type of oscillator circuit that incorporates an odd number of in- verters. Ring oscillators are particularly useful in soft robotic applications because they produce multiple phase-shifted os- cillating outputs, with the number of phases determined by the number of inverters (one per inverter). This enables the actuation of multiple actuators in a phased, sequential manner, which is common in soft robots.

The simplest fluidic ring oscillator circuit consists of three valves. Each valve is connected to a constant supply pressure through its inlet. The output of each valve is then connected both to the input of the next valve (its pouch) and to the ground (a leak to the atmosphere) (Fig. S3A). After an initial transient phase, the valves begin switching between open and closed states in sequence. At any given moment, one valve is open, a second is transitioning from open to closed, and the third is transitioning from closed to open. When the first valve turns off, the second transitions from closed to open, and the last transitions from open to closed.

This configuration has no stable state. Since each valve’s switching event disrupts the state of the next valve, the circuit continuously cycles through its states without settling into a stable equilibrium. This is because when any valve changes its state, it triggers the adjacent valve (whose input is connected to its output) to change state as well, ultimately leading to continuous state changes. As a result, the circuit produces three oscillating pressure signals, each occupying approxi- mately one-third of a full cycle (Fig. S3C).

Next, we connect one of the valve outputs to a fluidic unit configured as a sensor, as described in the previous section. Upon connecting the sensor, its intrinsic resistance amplifies the output node to which it is attached (Fig. S3D). This ef- fect can be highly beneficial in cases where different output amplitudes from a ring oscillator are desired. When the sensor is externally stimulated, it resists the flow proportionally, continuously varying the amplitude (Fig. S3B). This leads to amplitude amplification, which can exceed a 200% gain (Fig. 4E). Such an effect is useful in applications where introduc- ing a bias into one of the actuators is desired, as demonstrated in the following section.

## Controlled Soft-Actuated Shaker

In this section, we investigate two main aspects. First, how integrating fluidic units in different configurations can enable applications that respond to external stimuli. Second, we explore the potential of controlled ring oscillators to enhance applications that are typically based on ring oscillators. As a case study, we present a sorting application in which small beads are separated from a mixture of variously sized beads. The beads are sorted through three different holes based on a desired bias controlled by a ring oscillator circuit (Fig. S4A).

This application utilizes a total of nine fluidic units, with every three cells configured as valves, sensors, and actuators, re- spectively. The first set of valves forms a ring oscillator circuit, whose three outputs are connected to three sensors mounted on a sensor deck. These sensor outputs, in turn, drive the set of actuators (Fig. S4B).

The sorting setup consists of a sorting stage where the beads are initially grouped at the center. Only the small beads can escape the center container through three axisymmetric gates designed specifically to allow their passage. These gates are simple openings that match the size of the small beads. The sorting stage is positioned atop a base that houses the actua- tors.

The actuators are connected to the base by their fluidic unit bodies, while their tube ends are fixed to the table, anchoring the entire setup. This grounding restricts movement in the x, y, and z directions while allowing rotation around the x-axis. When the actuator pouches are inflated, the base and sorting stage tilt in the direction opposite to the activated actuator.

Since the three actuators are triggered sequentially due to the nature of the ring oscillator, the sorting stage tilts in a cir- cular motion, causing the beads to rotate within it (Mov. S3).

When the ring oscillator circuit is pressurized without any sensor stimulation, the beads rotate in a circular pattern and are randomly dispensed into the three containers (Fig. S4C). Upon repeated trials, no consistent preference for any specific container is observed. However, when one of the sensors is altered (by bending its sleeve), a bias is introduced in the cor- responding actuator. This occurs because the output amplitude is amplified, as explained in previous sections. As a result, the rotational movement of the sorting stage is skewed toward a specific dispensing container, directing most of the small beads into the desired outlet. This approach demonstrates how incorporating sensors into applications can enhance their functionality, making them more adaptive and responsive to external influences.

## Model of the Emergent Synchronization from Implicit Coupling of Units

To gain insight into how multiple coupled units synchronize (Fig. 3), we develop a basic Kuramoto model (37) of coupled self-oscillators. In particular, with a basic model, we aim to test the hypothesis that synchronization is a consequence of the implicit coupling (35) between the units, facilitated by the common robot’s body and by the shared substrate. More- over, we aim to capture how different arrangements of the units on the robot’s body affect the various distinct synchroniza- tion patterns that emerge. Can we predict the emerging phase shift between the units, based on their spatial arrangement on the body?

### Derivation of the Model

We model each unit as an oscillator *i* with natural frequency *ω_i_* and phase *θ_i_*. We assume each oscillator *i* to be implicitly coupled to all other *N −* 1 oscillators, because they are mechanically attached to the same body and share the same rigid substrate that they are in contact with. For generality, we assume the coupling strength to be heterogeneous among the units, as the relative orientation of each pair of units varies among pairs. Following these assumptions, the phase of each oscillator *i* follows the standard Kuramoto ordinary differential equation (ODE) (37; 36):

$\frac{d\theta_{i}}{dt}=\omega_{i}+\sum_{j=1}^{N} K_{ij}\sin\left( \theta_{j}-\theta_{i} \right),$ (S3)

where *K_ij_* is the coupling constant between oscillator *i* and oscillator *j* (Fig. S10A). The ODE expresses that the change in phase of each oscillator *i* at time *t* depends on the interactions with all other oscillators, with the sine term indicating that the phase of oscillator *i* is being pulled toward the phase of oscillator *j*. For *K_ij_ >* 0, the phases tend to be attracted to each other (*positive coupling)*, while for *K_ij_ <* 0, the phases tend to drift apart (*negative coupling*). The phases of pairs of oscillators with larger modulus of *K_ij_* will be more strongly attracted or repelled than pairs with smaller *K_ij_* modulus values.

Now, we pose a key assumption: the sign of the coupling between units *i* and *j* (positive or negative) depends on the rela- tive orientation between the two units on the robot’s body. This assumption stems from the following intuitions about our physical system. i) If two units are oriented parallel to each other and pointing in the same direction (Fig. S10B), then when unit ‘a’ is actuated, it will lift the shared body, hence easing the actuation of nearby unit ‘b’. In such conditions, the coupling is positive, as the actuating units are encouraging each other’s actuation. ii) If the two units are oriented in

a collinear way and pointing in opposite directions (Fig. S10C), when unit ‘a’ is actuated, it will lift the body, providing higher load to unit ‘b’ (through the robot’s body rotating) and hence reducing its actuation capability. Under such condi- tions, the coupling is negative, as the actuating units inhibit each other’s actuation.

Following this assumption, we can now formally remap the orientation angle between two units *β_ij_* to their coupling con- stant *K_ij_*. For simplicity, we decide to assign *K_ij_* = 1 when units *i* and *j* are positively coupled (parallel to each other, *β_ij_* = 0*^◦^*), and *K_ij_* = *−*1 when they are negatively coupled (opposite to each other, *β_ij_* = 180*^◦^*). In Figure S10D, we show the orientation angle*↔*coupling constant remapping, that is, values of *K_ij_* corresponding to various orientation angles *β_ij_* between the units. For units parallel to each other (*β_ij_* = 0*^◦^*), we have positive coupling (*K_ij_* = 1). For units opposite to each other (*β_ij_* = 180*^◦^*), we have negative coupling (*K_ij_* = *−*1). Note that for units oriented *±*90*^◦^* w.r.t. each other, the remapping gives *K_ij_* = 0, which matches the physical intuition about the system. In fact, in the ideal case of units ‘a’

and ‘b’ being perfectly orthogonal to each other, the actuation of unit ‘a’ should have no effect on the actuation of unit ‘b’: ideally orthogonal units are fully decoupled.

We can now generalize this remapping, as illustrated in Figure S10E. In general, given a circular (or polygonal) robot’s body plan, and given an orientation angle *β_ij_* between units *i* and *j*, the coupling constant between the two units is given by:

*K_ij_* = cos *β_ij_,* (S4)

which satisfies the cases in Figure S10D.

Note that, practically, we apply this general remapping to every pair of units arranged with an arbitrary relative angle around the circular (or polygonal) robot’s body plan. To give an example, in the relatively simple case of the arrangement in Figure S10A, we obtain *K*_12_ = *K*_21_ = *K*_23_ = *K*_32_ = *K*_34_ = *K*_43_ = *K*_14_ = *K*_41_ = 0 and *K*_13_ = *K*_31_ = *K*_24_ = *K*_42_ =

*−*1. Also note that, in practice, we assign non-identical natural frequencies to the *N* Kuramoto oscillators. The natural frequency of each oscillator is picked randomly from a normal distribution around 1 Hz with standard deviation 0*.*05 Hz. These values differ from those in the experimental case around 7 Hz. This choice of picking around 1 Hz is a simplification: given that the coupling strength is assigned as bounded within *±*1, the coupling would not be strong enough to synchro- nize oscillators at higher frequencies successfully. However, one could choose to apply a multiplying factor to the coupling constants, and the simulated oscillators would then synchronize even at the higher 7 Hz frequencies.

Once we have assigned the natural frequencies *ω_i_* of each oscillator and the relative orientation angle between each unit

pair *β_ij_*, we obtain the coupling constants *K_ij_* between each oscillator pair using Equation (S4). Then, we forward-integrate the ODE in Equation (S3) using the MATLAB solver ode45, with a time span of 60 s. We then extrapolate the average phase differences between each oscillator pair during the last 40 s of the simulation, to discard the initial transient. We consider the average phase differences as the main output of our model, to be compared with the experimental results in Figure 3.

### Results of the Model

First, using the model, we study a relatively simple case where the units are ideally positively and negatively coupled. We assume that the units are oriented on the robot’s body as two pairs: within each pair, units are parallel to each other (*β*_12_ = *β*_34_ = 0*^◦^*), and the two pairs are oriented opposite to each other (*β*_23_ = *β*_41_ = 180*^◦^*) (Fig. S11A). In this configu-

ration, the result is that oscillator 1 is in-phase with oscillators 2, as well as 3 with 4 (Fig. S11B): this is expected, as units within a pair are positively coupled. On the other hand, the pair 1-2 is oscillating anti-phase with the pair 3-4 (Fig. S11B): this is also expected, as the two pairs are assumed to be negatively coupled with each other. Importantly, these results of the model match well with the experiments in Figure 3G.

Then, we test the case where 5 units are arranged uniformly around the robot, with a 72*^◦^* angle between each pair (Fig. S11C).

The resulting phase differences between the units match the experimental results in Figure 3C: the units activate one after the other, with phase differences of approximately integer multiples of 72*^◦^* (Fig. S11D).

Note that, in Figure S11A-D, the similarity between the assigned orientation between the units (*β_ij_*) and the resulting phase differences is a coincidence arising from those specific configurations. To confirm this, we analyze an additional sce- nario where 6 units are arranged as two opposite triplets (with units within a triplet oriented at a 30*^◦^* angle) (Fig. S11E). The resulting phase differences show an interesting pattern: the two triplets are oscillating anti-phase with each other, and within the triplet, the oscillators are in-phase (Fig. S11F). These results indicate that the two triplets are behaving qual- itatively similar to the ideal case with two pairs of parallel units opposite to each other. This is because the units within

a triplet are positively coupled (although not ideally, with 0 *< K_ij_ <* 1). The units within the triplet are also negatively coupled with the units of the other triplet (although not all ideally, with *−*1 *≤ K_ij_ <* 0).

In conclusion, the model, despite its simplicity and abstraction, proves to be a powerful high-level tool for predicting how the units synchronize when implicitly coupled through the substrate. In particular, assuming a circular body plan, the model accurately predicts how the relative physical orientations of the units on the robot affect the synchronization pat- terns (the phases) of the oscillators.

## Movies Captions

**MOV S1. Airflow Blocking Mechanism** 3D Animation of sleeve and tube assemblage cross-section throughout the bending process. Upon bending the sleeve, the tube conforms on its inner wall blocking the flow.

**MOV S2. Multifunctional Unit configurations** The fluidic unit is shown and then configured into 4 different con- figurations; sensor, actuator, valve, and self-oscillating actuator. A schematic is displayed alongside every configuration showing the used ports and the neglected ones. Finally, the waveforms of pressure input/output are shown in every con- figuration in real time.

**MOV S3. Controlled Shaker** This shaker is based on a three-stage ring oscillator circuit. Each output of this circuit is connected in series to a fluidic unit configured as a sensor then to another configured as an actuator. Four cases of opera- tion are demonstrated, First, the idle case where the amplitude of the three outputs are similar, the shaker dispenses the small beads randomly as shown in the bar chart provided. Then, there are three cases when every sensor unit is physically actuated, amplifying the magnitude of the output it is connected to. This creates a different bias in every case, directing the small beads to be dispensed from a specific channel.

**MOV S4. The effect of the counterweight on the self-oscillating limb operation.** When the outlet of the flu- idic unit is connected to its input (pouch) and the supplied pressure is swept, the unit exhibits high-frequency oscillations.

However, these oscillations occur only within a narrow pressure range. When a counter-moment is applied to the tube of the fluidic unit, the oscillations become consistent across a broader pressure range, albeit at a reduced frequency. Slow- motion analysis reveals a repeating sequence comprising: (1) inflation of the pouch, (2) blockage of flow, and (3) deflation of the pouch followed by a reset of the tube.

**MOV S5. Multimodal Hoppers.** The self-oscillating actuators are employed as limbs in a soft-actuated hopper, where the counter-moment required for oscillation is provided by the weight of the hopper body. When these limbs are symmetri- cally arranged around the pentagonal base of the icosahedron structure, the hopper exhibits stationary jumping behavior. However, breaking this symmetry introduces a directional bias, causing the robot to hop forward.

**MOV S6. Crawler sensitive to the edge of terrain.** The versatility and multifunctionality of the fluidic unit enable the construction of robots with decision-making capabilities using only a single input and minimal hardware. By integrat- ing four units in distinct roles—a sensor, a valve, and two limbs—a crawler capable of detecting and responding to table edges can be realized. The sensor is held closed by the robot’s weight as it presses against the table surface. When the robot reaches an edge and the sensor is no longer supported, it opens, allowing air to flow to a safety valve. This valve then shuts off the air supply to the limbs, effectively halting the robot’s motion.


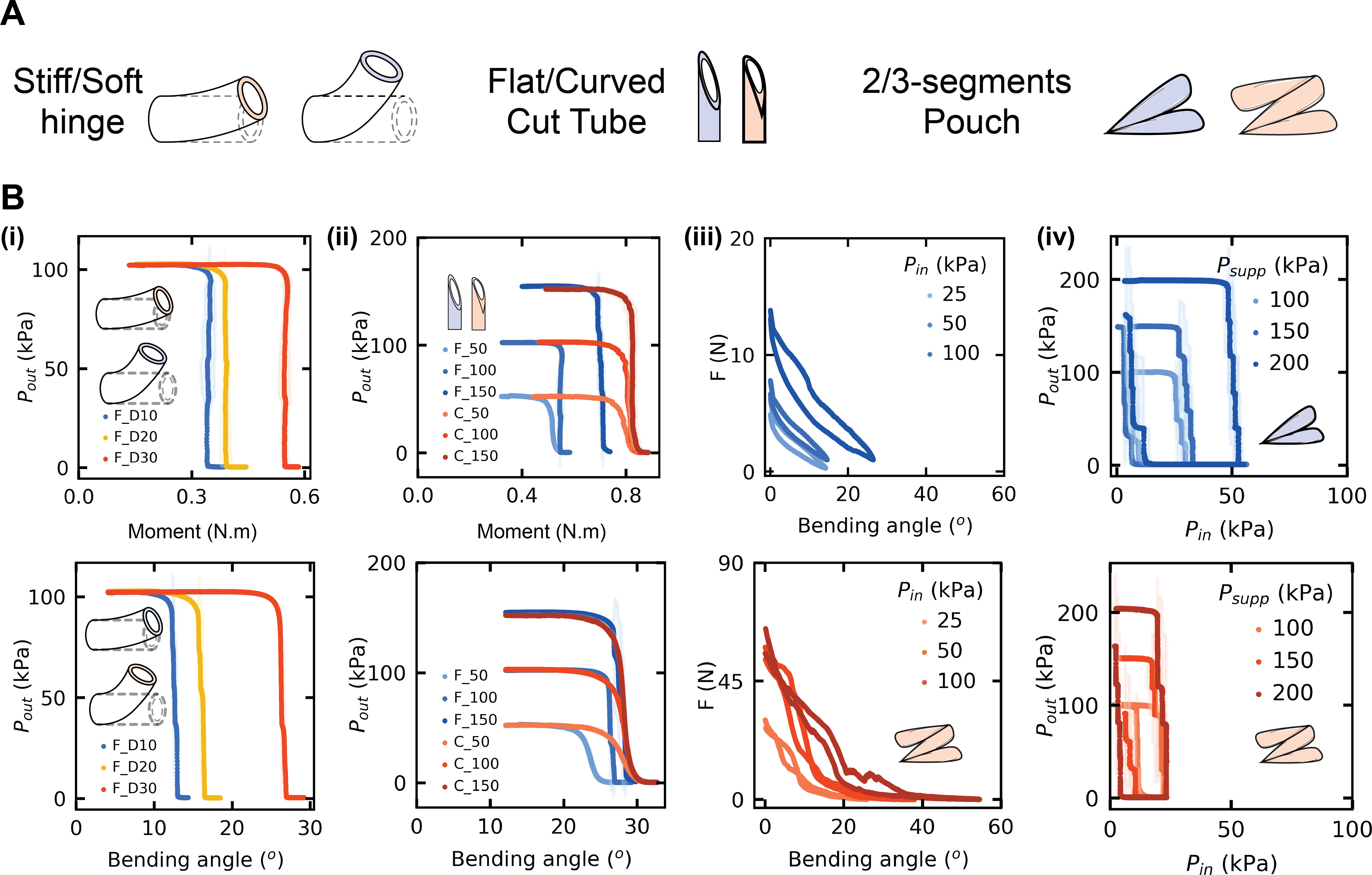


Figure S1: **Fluidic Unit Design Space.** (A) Key parameters influencing the unit’s performance including the sleeve ma- terial, the tube cut profile, and the number of segments forming the inflatable pouch. (B) The impact of these parameters on the unit’s behavior across different configurations—as a sensor, an actuator, and a valve. The shaded areas in panels (i), (ii), and (iv) indicate the standard deviation calculated over five trials.


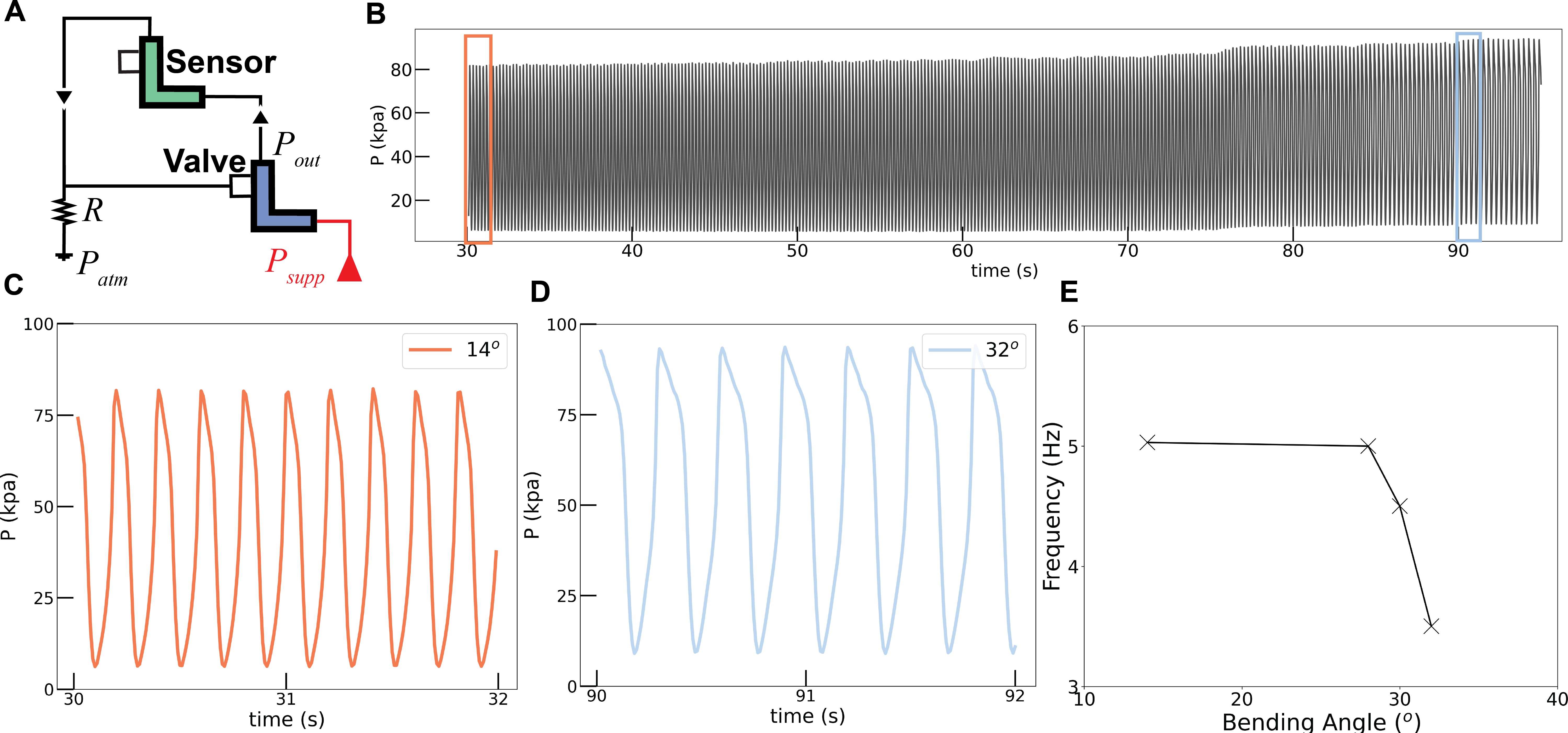


Figure S2: **Controlled relaxation oscillator characterization**. (A) The relaxation oscillator circuit with a sensor con- nected to its output. (B) Full stream of oscillating pressure upon changing its frequency using the sensor. (C-D) Snapshots of the frequency waveform at different bending angles. (E) The change in frequency on changing the sensor bending angle.


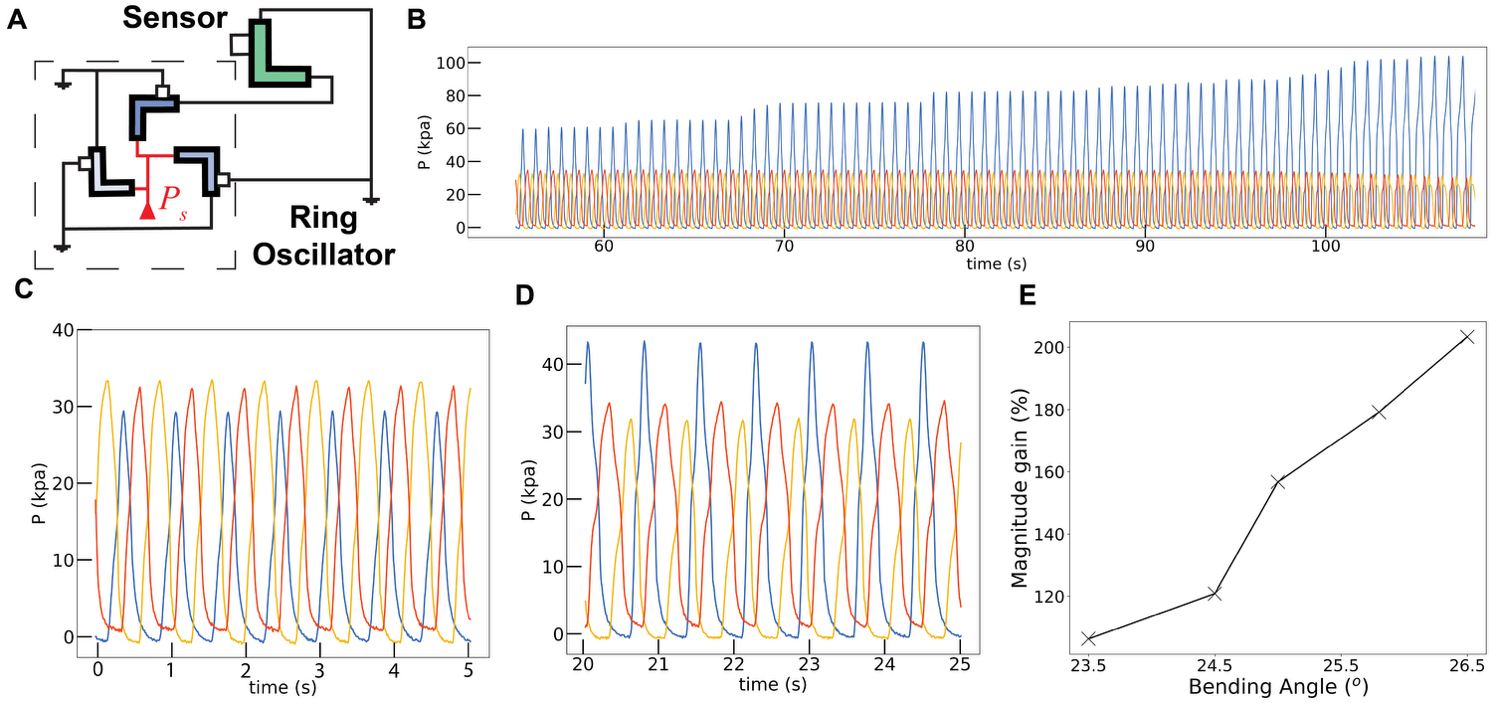


Figure S3: **Controlled ring oscillator characterization**. (A) The ring oscillator circuit schematic and the sensor connection to one of the outputs. (B) The full stream of data acquired for the circuit while changing the sensor bending angle. (C) The waveforms of the ring oscillator before connecting the sensor (D) and after connecting it showing the effect of its intrinsic resistance on the corresponding output. (E) The relationship between the sensor bending angle and the gain in magnitude.


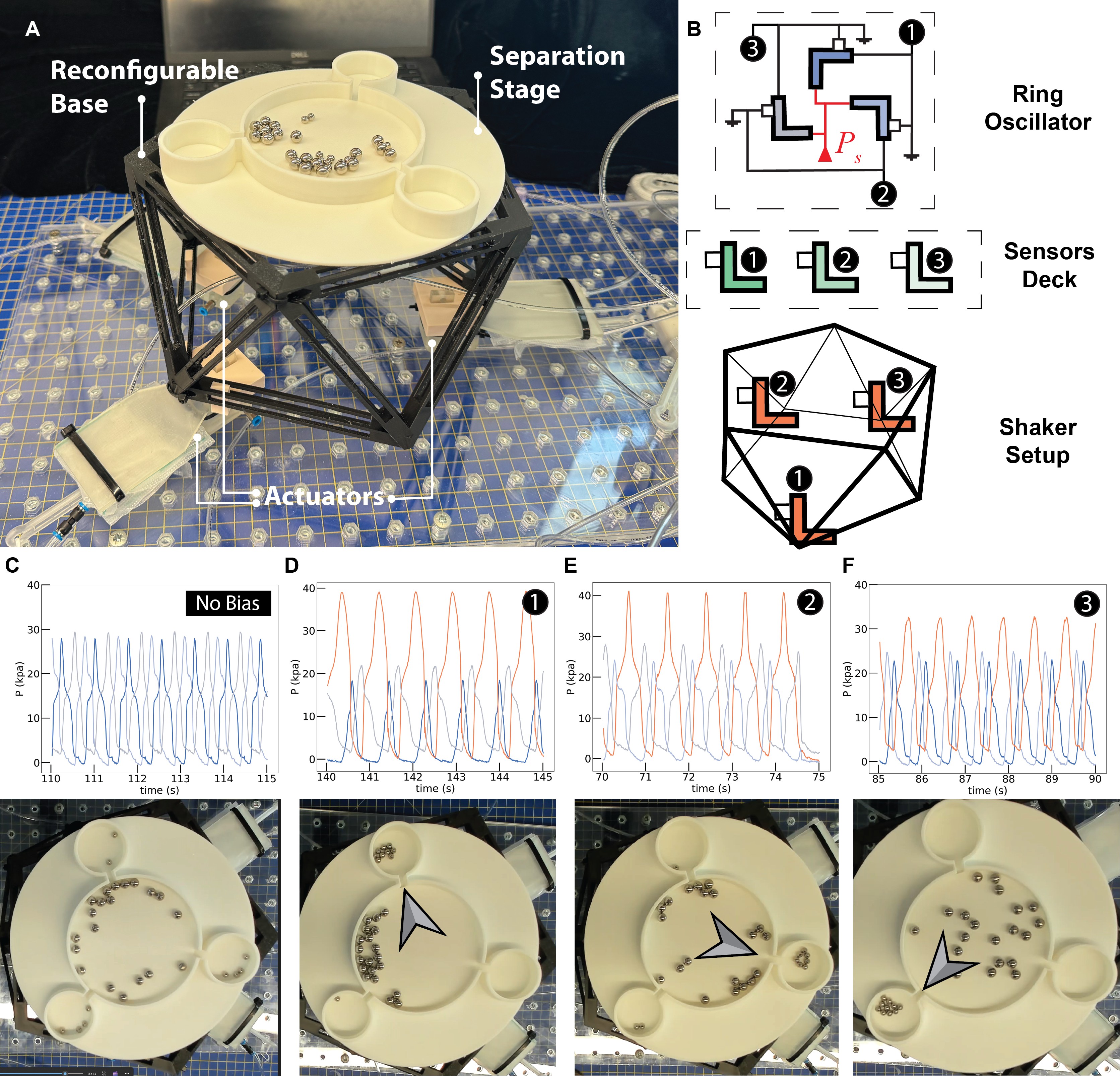


Figure S4: **Controlled Shaker.** (A) The shaker setup with its main components labeled. (B) The schematic of the circuit with outputs labeled (1-3) and their connections to the sensors and actuators. (C) The waveforms of the ring oscillator

at no bias from the sensors (D-F) and after implying a bias from each sensor and the corresponding result on the shaker dispensing direction.


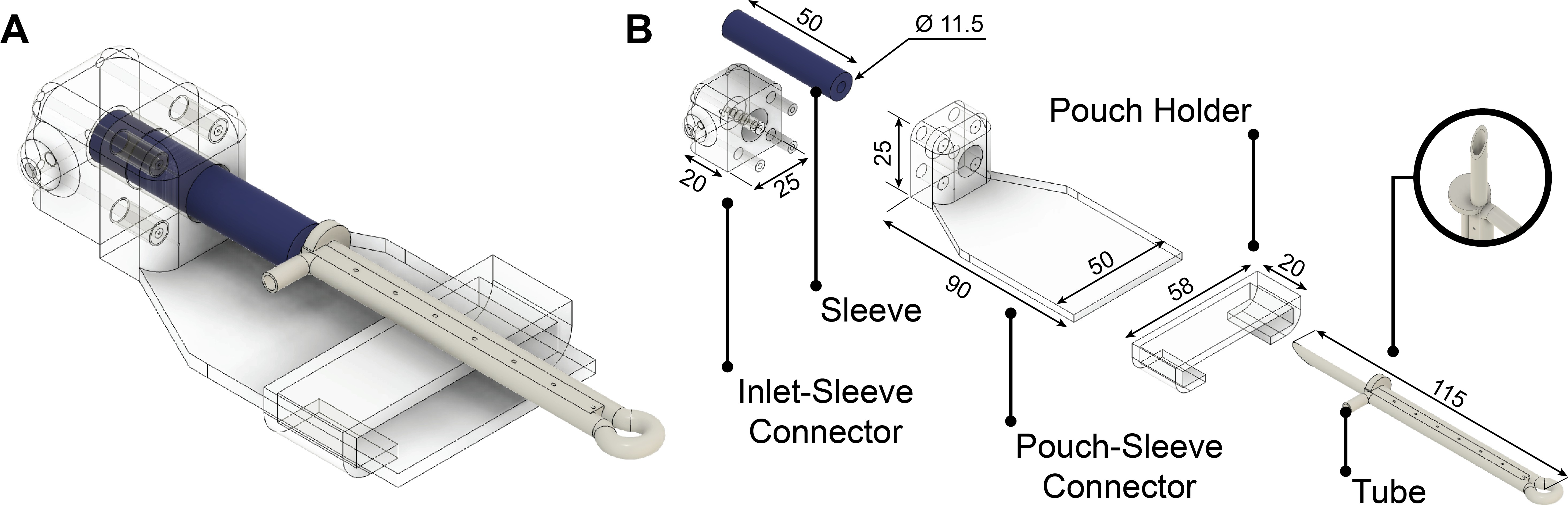


Figure S5: **Fluidic Unit Design.** (A) A rendered photo of the fluidic unit assembled. (B) Exploded view of the fluidic unit components and their dimensions.


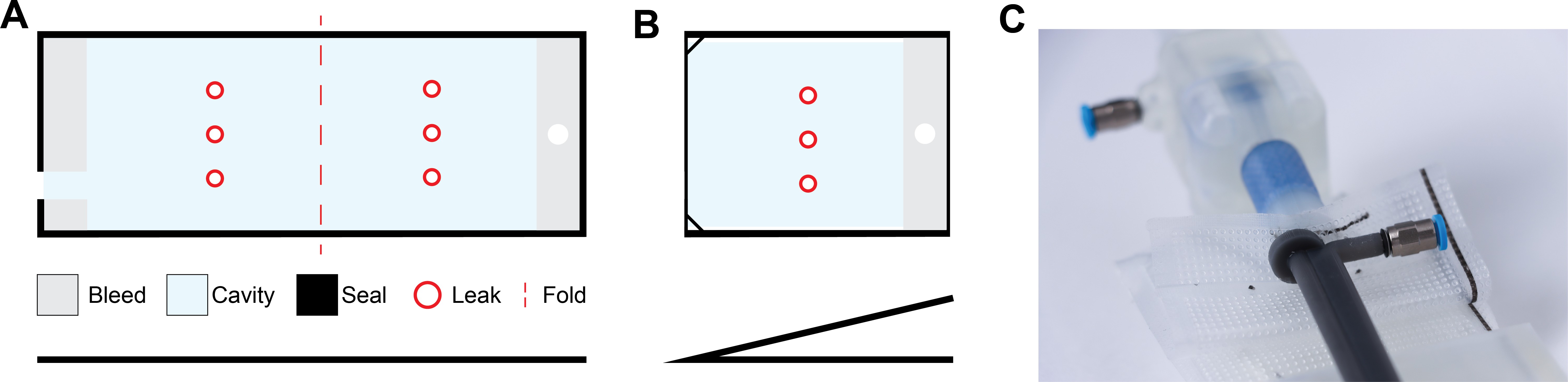


Figure S6: **Pouch Design.** (A) A 2D representation of a 2-segments pouch from top and front views. Each segment has 6 leak points (3 at each side of the segment). Two bleed areas are subtracted from the pouch total volume, one is created by the glue that seals the pouch from the input tube side. The other is added, so that we can add a hole that the fluidic unit tube can go through into the sleeve, making connection junction for the three components. (B) The two-segments of

the pouch after folding on top of each others. Two seal lines at 45*^o^* at the ends of the fold to maintaing this geometry after multiple actuation. (C) A photo of the junction where the outlet tube, the pouch, and the sleeve are connected.


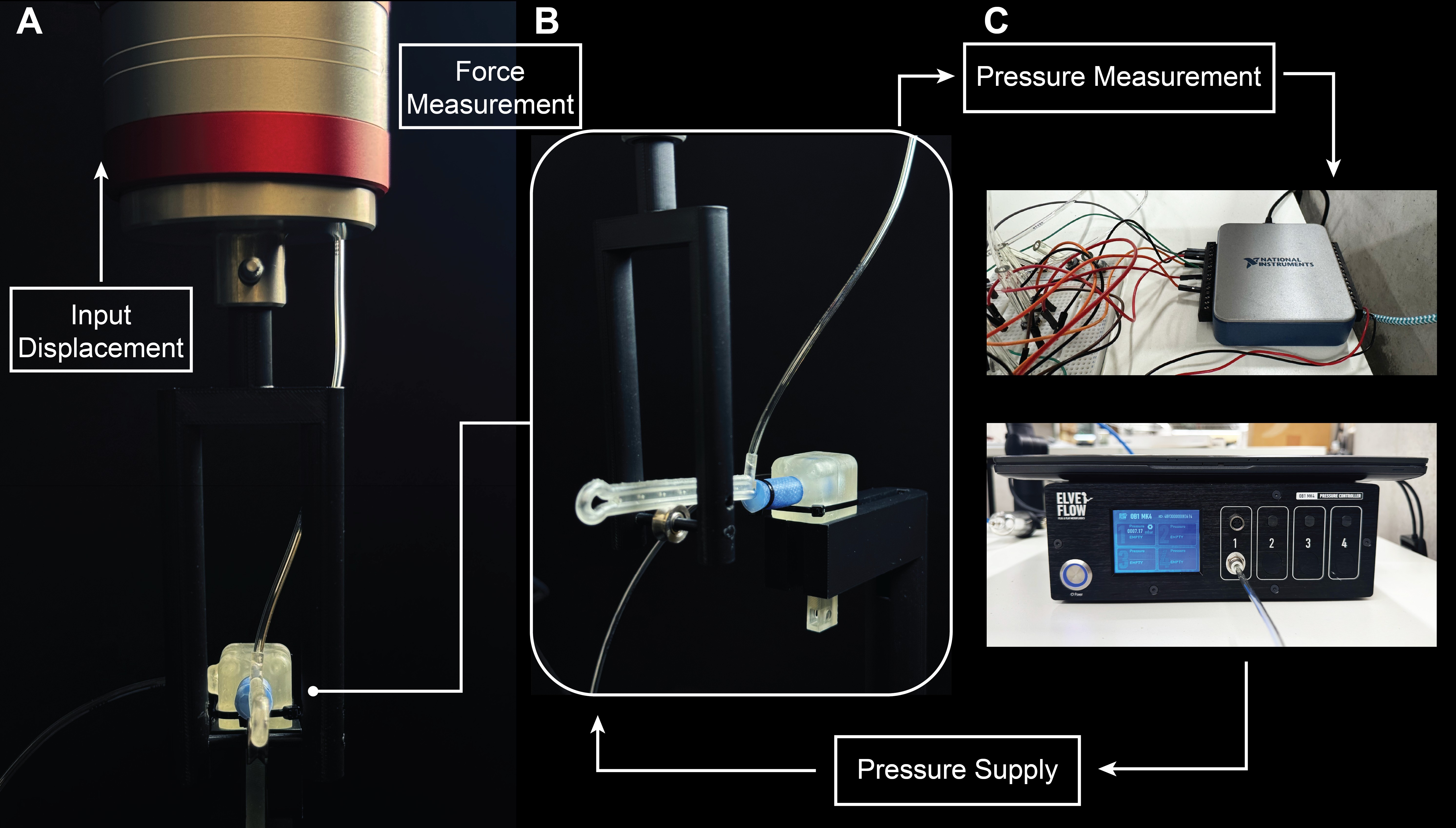


Figure S7: **Fluidic Unit characterisation.** (A) The experimental setup where the fluidic unit was characterised me- chanically. Input displacement was performed by the tensile testing machine head, and the resistance force of the sleeve bending moment was recorded against the change of pressure flowing from the ilet to outlet. (B) A zoomed view of the flu- idic unit, showing the custom 3D-printed holder where it is placed on, that allows shifting it back with respect to the head. This allows the outlet tube to be centered below the machine head. A fork-shaped attachement is added to the machine head, which includes a V-groove bearing at its end on which tube sits. Upon the start of the test the head moves upward, pushing against the tube at a fixed horizontal displacement with respect to the sleeve hinge. (C) The fluidic circuit work- flow; a pressure controller is used to supply the fluidic unit with constant pressure, the outlet tube is connected to pressure sensor and its data is logged with USB data acquisition device.


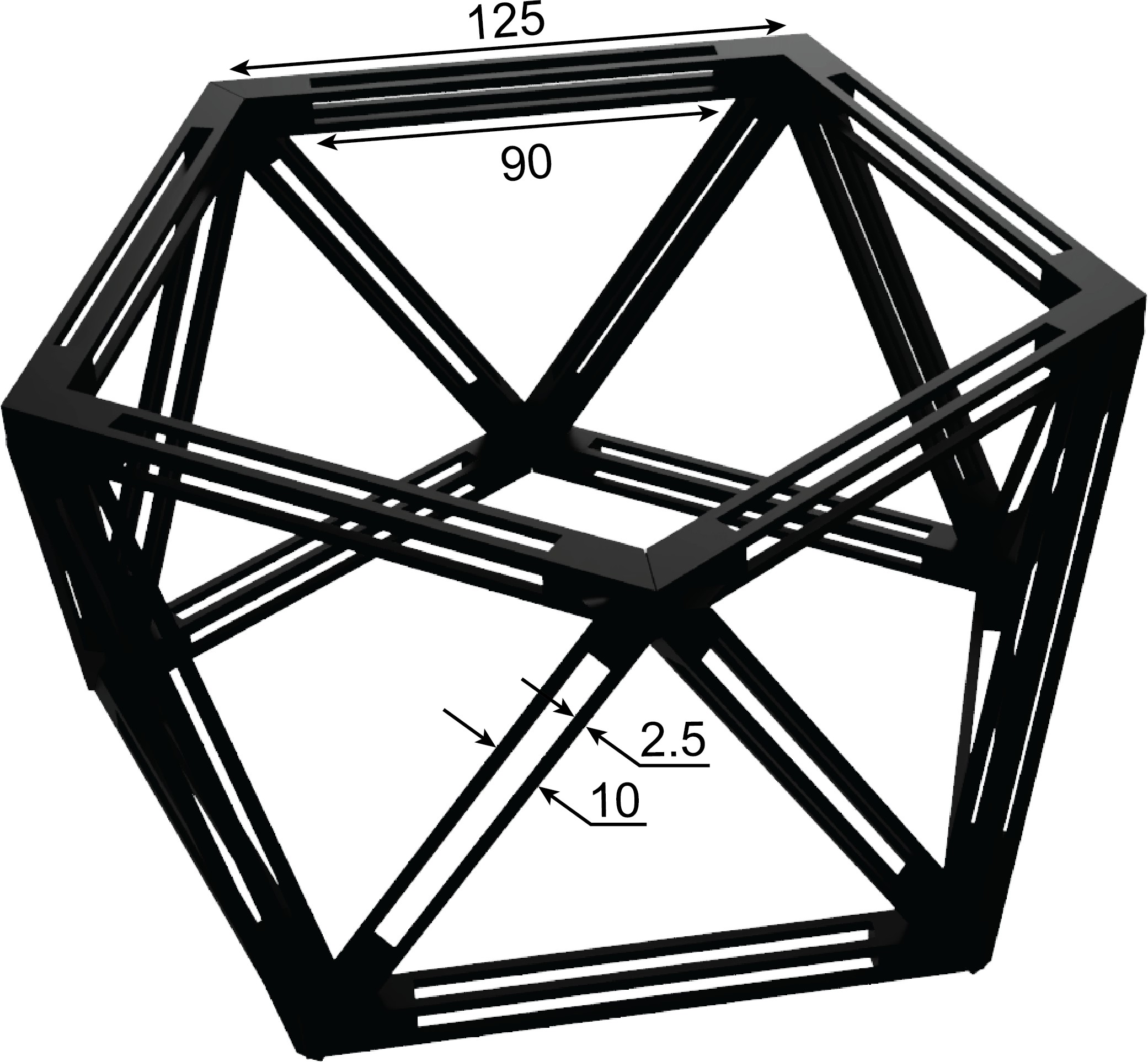


Figure S8: **Multifunctional Base.** The icosahedron structure used as Lego-like base for the fluidic units. The linkes of the structure has open slots to attach the fluidic units in different positions, creating all the different robots proposed in this work.


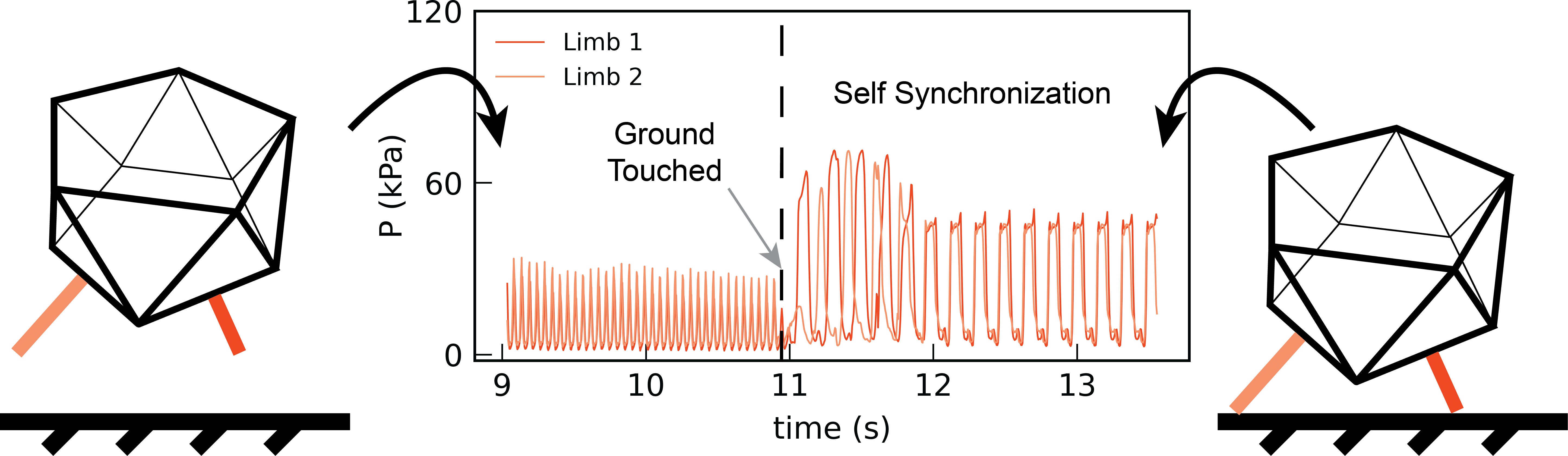


Figure S9: **Crawler limbs behavior.** Before touching the ground, the crawler’s self-oscillating limbs oscillate at high frequencies at a low amplitude. Upon touching the ground, the robot enters a transient state of random behavior, until the limbs passively self-synchronize, and then forward movement emerges.


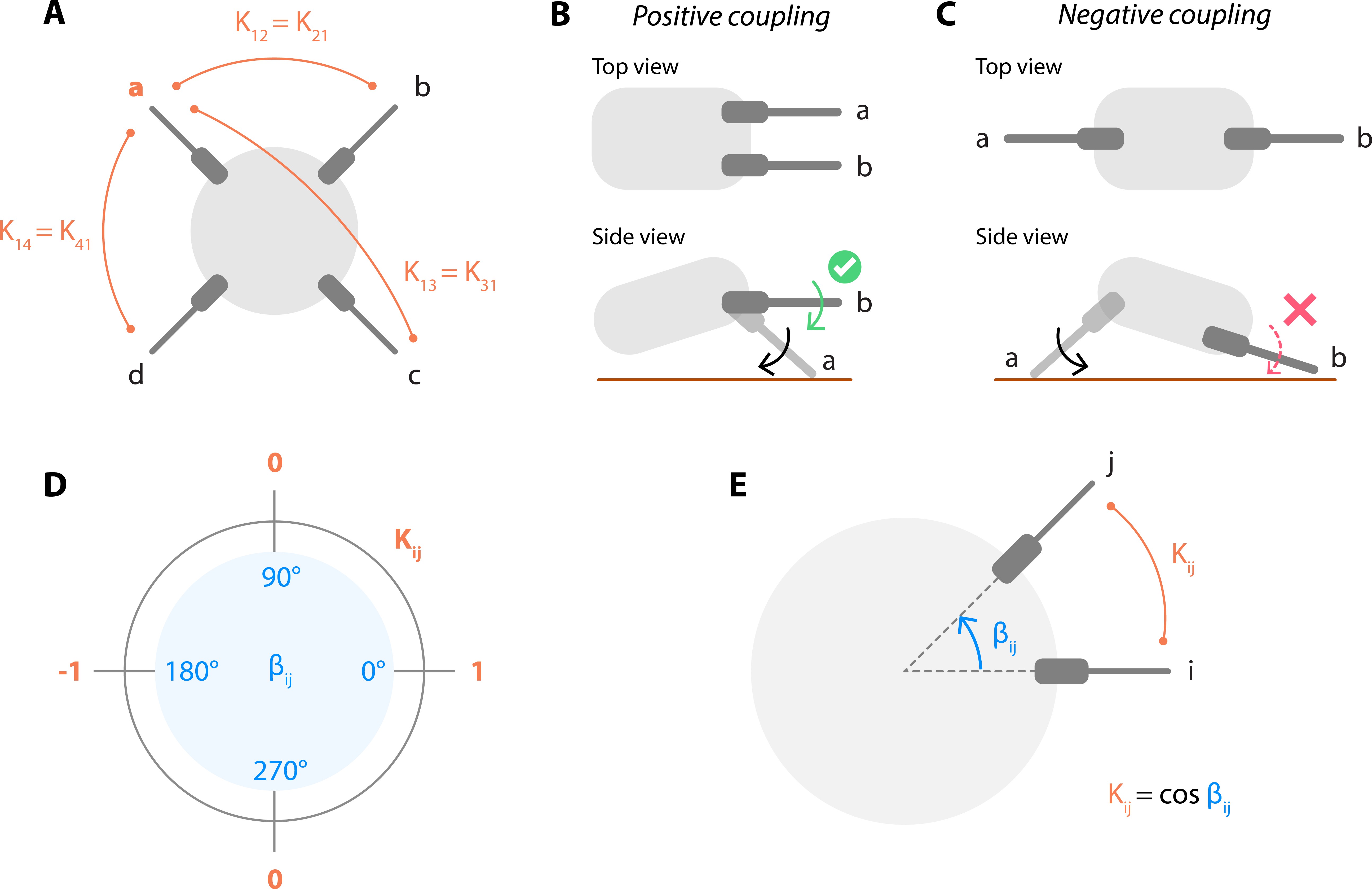


Figure S10: **Intuition behind the model of the implicit coupling.** (A) In an example case with four units, unit ‘a’ (orange) is coupled to all other units ‘b’, ‘c’, and ‘d’, and each pair has a distinct coupling term. (B) Among two units in a pair, we assume that when the units are parallel to each other, they exhibit ideal positive coupling, as the activation of

unit ‘a’ eases the activation of unit ‘b’ by lifting the shared body. (C) We assume that ideal negative coupling occurs when the units are rotated 180*^◦^* with respect to each other, as the activation of unit ‘a’ inhibits the activation of unit ‘b’, by rotating the shared body. (D) Following this intuition, we have a remapping from the angle *β_ij_* between units *i* and *j* and their coupling term *K_ij_*: *β_ij_* = 0*^◦^ → K_ij_* = 1, and *β_ij_* = 180*^◦^ → K_ij_* = *−*1. (E) This remapping leads to a general equation for *K_ij_* as function of *β_ij_*, that is *K_ij_* = cos *β_ij_*.


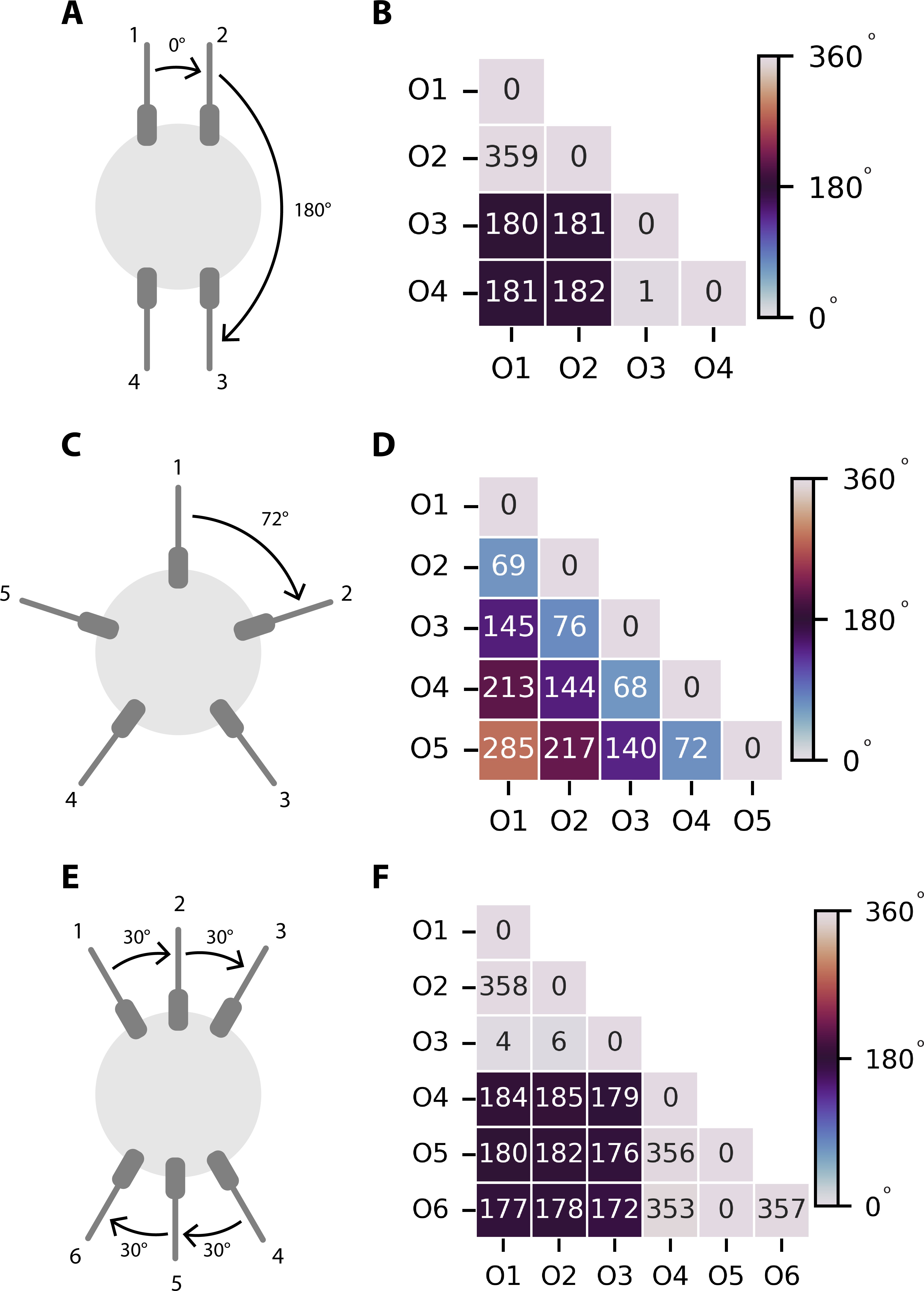


Figure S11: **Results of the model.** (A) Top view of the schematic of the modeled robot’s configuration with two oppo- site pairs of parallel units. (B) Resulting average phase difference between each unit for the configuration in A. (C) Top view of the schematic of the modeled robot’s configuration with five units uniformly arranged around the robot’s body, with a 72*^◦^* angle in between each pair. (D) Resulting average phase difference between each unit for the configuration in C. (E) Top view of the schematic of the modeled robot’s configuration with two opposite triplets of units. (F) Resulting average phase difference between each unit for the configuration in E.
